# BioMOF-Based Anti-Cancer Drug Delivery Systems

**DOI:** 10.3390/nano13050953

**Published:** 2023-03-06

**Authors:** Sandy Elmehrath, Ha L. Nguyen, Sherif M. Karam, Amr Amin, Yaser E. Greish

**Affiliations:** 1 Department of Chemistry, United Arab Emirates University, Al-Ain 15551, United Arab Emirates; 2 Department of Chemistry University of California—Berkeley, Kavli Energy Nanoscience Institute at UC Berkeley, and Berkeley Global Science Institute, Berkeley, CA 94720, USA; 3 Joint UAEU−UC Berkeley Laboratories for Materials Innovations, United Arab Emirates University, Al-Ain 15551, United Arab Emirates; 4 Department of Anatomy, United Arab Emirates University, Al-Ain 15551, United Arab Emirates; 5 Zayed Centre for Health Sciences, United Arab Emirates University, Al-Ain 15551, United Arab Emirates; 6 Department of Biology, United Arab Emirates University, Al-Ain 15551, United Arab Emirates

**Keywords:** metal–organic frameworks, BioMOFs, nanostructures, chemical modification, drug delivery, cancer treatment

## Abstract

A variety of nanomaterials have been developed specifically for biomedical applications, such as drug delivery in cancer treatment. These materials involve both synthetic and natural nanoparticles and nanofibers of varying dimensions. The efficacy of a drug delivery system (DDS) depends on its biocompatibility, intrinsic high surface area, high interconnected porosity, and chemical functionality. Recent advances in metal-organic framework (MOF) nanostructures have led to the achievement of these desirable features. MOFs consist of metal ions and organic linkers that are assembled in different geometries and can be produced in 0, 1, 2, or 3 dimensions. The defining features of MOFs are their outstanding surface area, interconnected porosity, and variable chemical functionality, which enable an endless range of modalities for loading drugs into their hierarchical structures. MOFs, coupled with biocompatibility requisites, are now regarded as highly successful DDSs for the treatment of diverse diseases. This review aims to present the development and applications of DDSs based on chemically-functionalized MOF nanostructures in the context of cancer treatment. A concise overview of the structure, synthesis, and mode of action of MOF-DDS is provided.

## 1. Introduction

**Figure 1:**
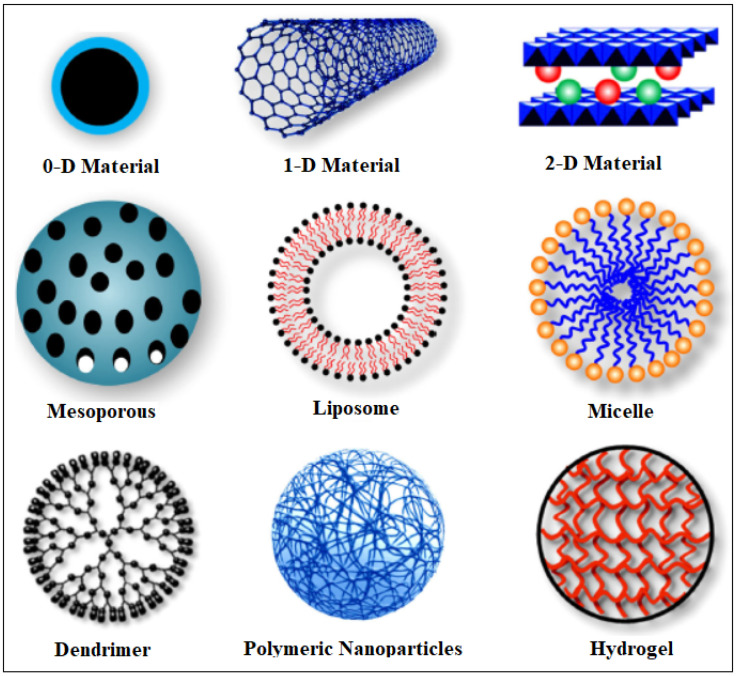
Different types of nanocarriers used as controlled delivery vehicles for cancer treatment. Reproduced with permission from Ref. [4]. Copyright © 2018, Nature.

Nanoparticles (NPs) are employed as delivery vectors for therapeutic payloads, enabling targeted and regulated transportation deep within bodily tissues [1]. TThis innovative technology holds great promise for numerous chemotherapeutic agents with poor pharmacokinetic profiles, swift clearance rates, and non-specific biodistribution [2]. Efforts in designing drug delivery carriers to overcome these challenges are made by improving targeted delivery, reducing toxicity and increasing drug effectiveness in targeted tissue. NPs-based DDSs can help overcome the challenges faced with conventional drugs. Drug nanocarriers are highly valuable because of their small size, high porosity, enormous surface area and tunable properties. Nanomaterial encapsulated drugs can also allow for controlled and sustained drug release of less soluble and poorly absorbed drugs. However, the efficacy of these vehicles depends on their hydrophobicity, size, shape and surface features [3,4]. Designing a bio-friendly material is essential for biological applications such as drug delivery. Ideally, the drug carrier should be biocompatible and biodegradable with minimum side effects. Various types of nanocarriers have been developed, such as metal NPs [5,6,7,8], micelles [9,10], liposomes [11], dendrimers [12,13] and hydrogels [14,15] (Figure 1 [4]). 

What differentiates these nanomaterials from each other are their surface properties, porosity, hydrophobicity, etc. For example, micelles, which consist of hydrophobic and hydrophilic components, can be tuned to release a drug in an environment upon the presence of stimuli (e.g., pH and enzyme). They can be extensively functionalized than other drug delivery vehicles because of their higher surface area owing to their nanosize. A micellar system, prepared by Watanabe et al., bearing camptothecin was conjugated with polymeric micelles composed of various poly(ethylene glycol)–poly(aspartate ester) block copolymers. This system showed prolonged circulation and efficient release of the drug at the tumor site [16]. Dendrimers have also been used as drug delivery vesicles in tumor tissue because of their modifiable peripheral groups and their ability to perform controlled and targeted drug delivery. They allow for a higher stability, increased half-life and bioavailability of drugs. Particularly, dendrimer conjugated drugs reduce systemic toxicity and increased accumulation in tumor tissue [17,18]. Liposomes, amphiphilic molecules composed of natural or synthetic lipids, were conjugated with doxorubicin (DOX), by Ogawara et al., and investigated in mice with colon cancer. The polyethylene glycol-coated liposomal DOX showed antitumor effects on DOX-resistant and non-DOX-resistant C26 cells [19]. The success of these NP systems has led to further investigation and development of anti-cancer carriers with increased efficacy and reduced toxicity. MOFs can overcome limitations faced with using conventional NPs-based DDS such as toxicity, poor bioavailability, burst drug release, particle aggregation and small drug loading capacity.

Previous reviews discuss the importance of biocompatible MOFs in biological applications and in biomedicine. In this review, we dive deep into the factors that determine the biocompatibility of a MOF structure such as the metal ion and organic linker constituents. Moreover, we discuss the various MOF synthesis methods and the ability of how these reticular materials can respond to certain intrinsic and extrinsic triggers. Understanding the response to certain stimuli will allow researchers to study MOFs for specific cancers and other diseases. Finally, the review focuses on all cancers that have exhibited success with MOF DDSs and/or MOF stand-alone treatments. 

## 2. MOFs and Their Biomedical Applications

MOFs are a class of crystalline materials with ultrahigh porosity and surface area that can exceed 6000 m^2^ g^−1^ [20]. These MOFs consist of metal ions or clusters coordinated to organic bridging ligands [21] allowing for the fine tuning and flexible design of pore size, surface area and functionality with different building blocks [22]. MOFs can be constructed in the form of 1D, 2D and 3D structures for a wide range of applications [23]. Their synthesis takes place by self-assembly of the metal ion cluster and the organic linker forming highly thermal and mechanical stable compounds [24]. The high crystallinity of MOFs provides defined networks and clear structural information, which is important in determining the interactions with guest molecules. Due to the multiple features of MOFs, they have been used in various applications such as gas adsorption, storage of clean gas fuels, catalysis, separation science and drug delivery [25,26]. Depending on the structure of the MOF as well as its desired application, there are various approaches to MOF synthesis such as solvothermal, hydrothermal, vapor diffusion, microwave synthesis, ultrasonic, mechanochemical and electrosynthesis. Moreover, MOFs can also be post-synthetically modified, introducing functional groups to allow for additional functionality while maintaining the MOFs integral network [27]. Due to the above-mentioned unique characteristics of MOFs, they have been also investigated for biomedical applications, especially in the areas of imaging, bio-sensing, bio-catalysis, drug delivery and cancer treatment [28,29,30,31]. In particular, MOF DDSs have been investigated for the treatment of breast [32,33,34,35,36,37,38,39,40,41,42,43,44,45,46,47,48,49,50,51,52,53,54,55], lung [52,53,56], oral [57,58], hepatic [59,60,61,62,63,64,65], pancreatic [66,67], colon [68,69,70,71,72], bladder [73], ovarian [74,75,76,77,78,79,80,81,82], cervical [71,83,84], brain [85] and blood cancers [86]. 

MOFs used for biomedical applications are often called “BioMOFs”. The origin of this abbreviation; BioMOF, was discussed by Cai et al. in 2019 [87]. Based on their hypotheses, a BioMOF is a MOF whose all or part of the organic linker is biological in nature, but with chelating characteristics [88,89,90]. A BioMOF whose organic linker is entirely biological in nature is abbreviated as Bio-MOF-xxx, where xxx refers to a number given to the BioMOF upon its synthesis and registration [91,92,93]. On the other hand, a BioMOF could be related to a regular biocompatible MOF that is feasible to act as a carrier of biological molecules and/or drugs [29]. The current critical review, therefore, describes in more details the highly established potential of BioMOF nanostructures, both pristine and chemically modified, as successful DDSs for the treatment of cancer. BioMOFs are intrinsically biocompatible with little to no toxicity. Few factors are to consider when selecting a metal to be used for biomedical applications including the kinetics of degradation, biodistribution, accumulation in tissues and organs and daily dosage requirements. These characteristics are often studied on the BioMOFs’ precursors; metal ions and the organic linkers, as well as on the BioMOFs made thereafter. 

### 2.1. Biocompatibility of Metal Ions

The lethal dose (LD_50_) of a substance or material is the dose it takes to kill half of the members of a test population. With MOF preparations, the most fitting metals with acceptable toxicities include Ca, Cu, Fe, Ti, Zn or Mg with a LD_50_ ranging from 0.025 g/kg to 30 g/kg. However, daily dosage and chemical formulation of the metal needs to be taken into consideration when using MOFs for biomedical applications [94,95]. Grall et al. evaluated the cytotoxic effects of MIL-100(Fe, Al, Cr) NPs on four human cell lines (A549, Hep3B, HepG2 and Calu-3) [96]. At the highest concentration of 64 μg/cm, MIL-100(Fe, Al, Cr) NPs did not induce toxicity in the A549, HepG2 and Calu-2 cell lines. A toxic effect was observed with MIL-100(Fe) in the Hep3B cell line, which could be due to the absence of TP53 expression in the hepatocarcinoma cell line. Further, biological applications generally require a much lower dose than what is used in most cytotoxic studies. 

MOFs can be selective and cytotoxic towards certain cell lines, making them successful DDSs. A Zr-based multivariate (MTV) MOF (UiO-66) was loaded with an anti-cancer drug and tested on MCF-7 breast cancer cells and HEK293 kidney cells. The MTV-MOF exhibited a drastic increase in cytotoxicity towards the breast cancer cell line and selective biocompatibility towards the HEK290 kidney cells (up to 1 mg/mL) [97]. The selectivity of MOFs will be further discussed in this review along with their ability to respond to certain intrinsic and extrinsic triggers.

### 2.2. Biocompatibility of Linkers

**Figure 2:**
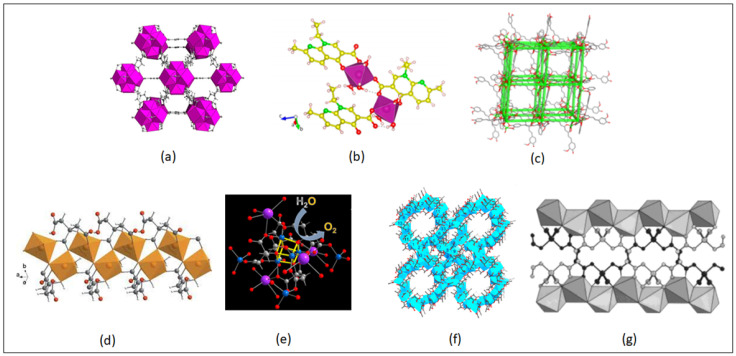
Various types of MOF structures for drug delivery formulations. (**a**) Zr-fumarate. Reproduced with permission from Ref. [100]. Copyright © 2019, MDPI. (**b**) Zn-nalidixate. Reproduced with permission from Ref. [107]. Copyright © 2019, American Chemical Society. (**c**) [Fe(H_2_cit)(H_2_O)]n (NICS-2). Reproduced with permission from Ref. [109]. Copyright © 2013, Wiley. (**d**) Co_4_O_4_ cubane. Reproduced with permission from Ref. [110]. Copyright © 2017, American Chemical Society. (**e**) MOF-1201. Reproduced with permission from Ref. [111]. Copyright © 2017, American Chemical Society. (**f**) K_2_Co(C_4_H_4_O_4_)_2_. Reproduced with permission from Ref. [112]. Copyright © 2001, American Chemical Society.

Exogenous linkers, polycarboxylic and imidazolate, have been proven to have low toxicity due to their high polarity and clearance under physiological conditions [94]. These linkers can also be functionalized to improve their pharmacokinetics and to allow for an improved delivery system of bioactive molecules. The use of functional groups has not only enhanced host-guest interactions but also the adsorption and delivery of the bioactive molecules due to changes in the MOF flexibility [98,99]. Alternatively, endogenous linkers with less toxic effects are also ideal for biomedical applications. A functionalized Zr-fumarate MOF has been reported to outperform UiO-66 for nanoscale drug delivery, as shown in Figure 2a [100].

Active pharmaceutical ingredients (APIs) have also been used as linkers to reduce adverse effects once the MOF components are degraded [28]. Using an API in the MOF structure can lead to improved dosage and/or solubility [101]. Miller et al. built a MOF using non-toxic iron and the therapeutically active linker, nicotinic acid. This bioactive MOF displayed a high drug uptake (71 wt%) and a fast release of the drug in phosphate buffer solution [102]. Zinc and bismuth were also used as coordination networks where an iron overload drug, deferiprone, was used as a chelating ligand [103,104]. Olsalazine, a prodrug of the anti-inflammatory 5-aminosalicyclic acid, has the same coordinating functionalities as 4,4′-dioxidobiphenyl-3,3′-dicarboxylate but with a longer chain length [105,106]. Other APIs have been used as MOF linkers including the antibiotic nalidixic acid and the anti-inflammatory, anti-oxidative and anti-cancer drug, curcumin (CCM) [107,108]. Medi-MOF-1 was initially synthesized solvothermally using the less toxic zinc as a metal node and CCM as a functional natural linker [108]. The MOF was loaded with ibuprofen (IBU) and used as a co-delivery cargo for the treatment of pancreatic cancer cells (BxPC-3) [108].

Citric acid and lactic acid are all biocompatible linkers that have also been used for synthesizing bioMOFs [109,110,111,112,113]. In 2013, the first neutral ferrous citrate MOF was synthesized hydrothermally and the structure was confirmed using single-crystal x-ray diffraction. Its structure consists of a pseudo-three-dimensional framework constructed from infinite chains of iron (Fe) polyhedra. The Fe nodes are hexa-coordinated by two partially deprotonated citrates and water molecule in a distorted octahedral manner [109]. Yang et al. constructed two porous chiral MOFs, MOF-1201 and MOF-1203, from Ca^2+^ ions and L-lactate [CH_3_CH(OH)COO^−^], where Ca^2+^ ions are bridged by the carboxylate functional group of the lactate and acetate linker, as well as the hydroxyl functional group of the lactate linker. This biocompatible Ca^2+^ MOF was synthesized using non-toxic lactate linkers where 1 L of water can degrade 120 ± 10 g of the MOF-1201, making it useful for medicinal purposes [111]. One of the final products of the Krebs cycle, L-malic acid, was used to synthesize homochiral MOFs with metals: Mn, Ni, Co, Cu and Ca [114,115,116,117,118]. The calcium MOF [Ca(HL-MA)]_n_ (H_3_L-MA = L-malic acid) has been solvothermally synthesized and displayed very high thermal stability which was determined by thermogravimetric analysis (TGA) and powder x-ray diffraction (PXRD) [118].

Peptides can also be considered as ideal candidates for synthesizing MOF structures as they can act as therapeutic agents. A tripeptide (glycine-glycine-L-histidine) and zinc 3D chiral MOF, ZnGGH, was synthesized and the behavior of this MOF was observed after inducing various chemically triggered conformational changes [119]. The functionalized internal surface and the flexible Gly-Gly part of the tripeptide increased the responsiveness of the ZnGGH framework to various solvents, controlling the chemical function of the MOF. Dioxane and cyclopentanol were not initially adsorbed by DMSO-solvated ZnGGH (DMSO stands for dimethylsulfoxide); however, the exchange of DMSO solvent from the pores with dimethylformamide (DMF) triggered the adsorption of these guests [119]. Solvent exchange helps activate the MOF structure while maintaining its integrity. It is usually carried out by replacing a higher boiling point solvent (used during synthesis) by a lower boiling point solvent [120].

## 3. Synthesis of BioMOFs

**Figure 3:**
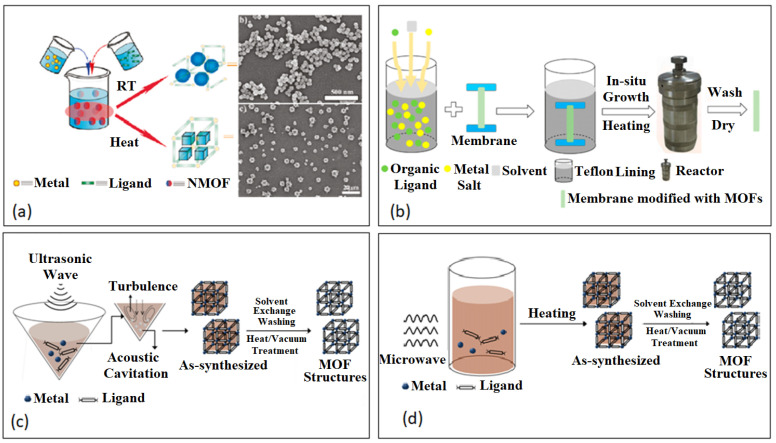
Various methods of MOF synthesis. (**a**) Low temperature synthesis. Reproduced with permission from Ref. [121]. Copyright © 2011, American Chemical Society. (**b**) Solvothermal/hydrothermal. Reproduced with permission from Ref. [122]. Copyright © 2018, MDPI. (**c**) Sonochemical. Reproduced with permission from Ref. [123]. Copyright © 2013, Springer. (**d**) Microwave-assisted solvothermal synthesis. Reproduced with permission from Ref. [123]. Copyright © 2013, Springer.

In a typical MOF synthesis, the precursor solutions are usually mixed together to allow for nucleation and crystal growth. Mixing of MOF precursors takes place in a suitable solvent at room temperature, while the formation of MOF takes place under various temperatures and pressure [121]. Methods such as solvothermal [122], sonochemical [123], electrochemical [124], microwave-assisted [125], vapor diffusion [126] and reverse-phase microemulsions [127] have been used to synthesize BioMOFs, as illustrated in Figure 3. Details of these methods are explained below. The key to obtain formulations that are stable and reproducible is to control the particle size of NPs as this dictates the properties of the NP’s, such as reactivity, external surface and packing [128,129,130,131]. When synthesizing NP’s for specific administration routes, the goal is to produce nanosized, homogenous, monodispersed and stable particles that fit for the targeted route [94]. 

### 3.1. Solvothermal/Hydrothermal

Conventional synthesis of MOFs takes place in a solvent at temperatures ranging from room temperature to around 250 °C [132]. Solvothermal and hydrothermal methods allow for a higher yield and smaller, more homogenous crystals than non-thermal methods [133]. Various parameters can affect the nucleation and growth of the MOF particles, such as temperature, reaction time and stoichiometry. Zinc imidazolate (ZIF-8) is an example of how reducing temperature and reaction time can produce nanocrystals with the size ∼85 nm with enhanced thermal, hydrothermal and solvothermal stabilities [134]. Horcajada et al. tuned porous hybrid solids such as MIL-88A and MIL-88B to improve their structures and porosities for better drug interactions and high loadings and to serve as nanocarriers for delivery and imaging applications. They determined that non-toxic porous iron(III)-based MOFs with engineered cores and surfaces were efficient drug nanocarriers for the delivery of anti-tumor and retroviral biomolecules [125]. 

### 3.2. Sonochemical

Recent MOF synthesis has been geared to a ‘greener’ approach. Using a synthetic method can minimize the use of organic solvents, decrease reaction temperature/pressure and reduce reaction time. Sonochemistry, electrochemistry and ball milling are a few examples of green synthesis. The interaction of high-energy ultrasound with the liquid sample leads to extremely high temperatures and pressures contributing to a rapid heating and cooling rate and ultimately fine crystal growth [132,133]. Li et al. synthesized HKUST-1 crystals under ultrasonic irradiation at ambient temperatures for 5–60 min [135]. They observed improved pore volumes and no significant differences in porosity when compared with the traditional solvothermal synthesis of HKUST-1. This work proves that environmentally friendly and efficient alternative methods to MOF synthesis are promising. 

### 3.3. Electrochemical

Electrochemical synthesis allows for the possibility of preparing a higher solids content when compared to batch reactions [132]. Bio-based MOF, [Zn_3_(BTC)_2_] (BTC = benzenetricarboxylate) was prepared using electrochemistry and sonochemistry and then loaded with IBU [124]. The electrochemical method produced larger average particle size (ca. 18.43 ± 8.10 µm) compared to the sonochemical method (average particle size ca. 87.63 ± 22.86 nm). The study also showed that the longer the reaction time took place under ultrasonic irradiation, the larger the MOF particle size [124].

### 3.4. Microwave-Assisted

Microwave-assisted synthesis involves the interaction between electromagnetic waves and moving electric charges in the MOF solid sample. Compared to conventional heating, microwave-assisted synthesis is faster and produces smaller crystals [132]. This rapid method is environmentally friendly and produces a high-yield of sample with good monodispersity and controlled size [133]. Horcajada et al. synthesized an iron terephthalate MOF, MIL-53 (Fe), at 220 °C for 30 min under microwave irradiation at 600 W yielding a flexible with framework with pore size of 8.6 Å and particle size of 350 nm [125]. The iron (III)-based MOF was successfully loaded with pharmaceutical drugs including, busulfan, azidothymidine triphosphate, ibuprofen, caffeine, urea and benzophenone. 

### 3.5. Vapor Diffusion

Vapor diffusion is a method that requires a small amount of reactants yielding good crystals with control over reaction parameters [136]. Cyclodextrin MOF (CD-MOF) is prepared using edible ingredients including, γ-cyclodextrin, potassium chloride and ethanol under vapor diffusion synthesis [126]. This porous “edible” MOF has been successfully used for various applications including drug delivery [137,138]. 

### 3.6. Reverse-Phase Microemulsions

This method allows for the control of the MOF particle size by tuning the dimensions of the micelles of a surfactant. A manganese-based BioMOF was synthesized using this approach and was further coated with a thin silica (SiO_2_) shell to enhance its stability in solution [127]. The Mn-based BioMOF showed a dual function in the diagnosis and targeted delivery of drugs with a controlled release ability [127]. 

## 4. Surface Modification of BioMOFs

**Figure 4:**
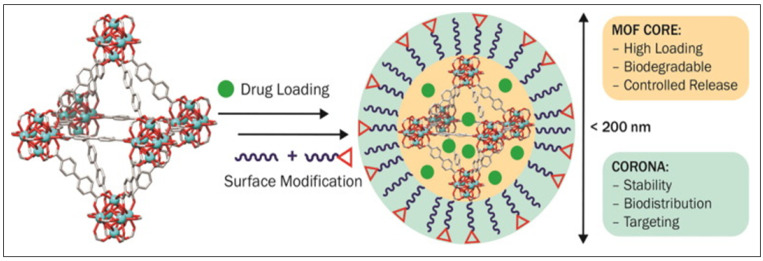
Surface modification of MOF nanostructures. Reproduced with permission from Ref. [140]. Copyright © 2018, American Chemical Society.

Surface modification of BioMOFs is a strategy that can solve challenges such as targeted delivery, opsonization by blood proteins, biodistribution and transcytosis of drug molecules [94], as illustrated in Figure 4. It can improve the MOFs water dispersity and reduce plasma protein binding to help avoiding the reticuloendothelial system and allow for targeted cell delivery of drugs [139]. Figure 4 shows the ability to modify the surface (internal and external) of the MOF structure in order to accommodate the differences in the hydrophobicity/hydrophilicity of the drug and MOF crystal [140] and hence improves its ability as a BioMOF DDS. 

Certain coating materials such as polyethylene glycol (PEG), can ‘protect’ the NMOF from early degradation through the development of a brush-like shell onto the surface of the NMOF; hence, sterically protect it from macrophage uptake [94]. This, in turn, allowed for a more targeted and increased accumulation of the biomolecule. MIL-100 (Fe) was functionalized with acryl-PEG (480 Da, 2 kDa and 5 kDa) and acryl-Hyaluronic acid (HA)-PEG moieties using a green, biocompatible and simple GraftFast method [141]. This method produced homogenous coatings and improved its shielding effect. The modified PEG-coated MIL-100 (Fe) produced a lower immune response while maintaining the drug loading and release. Additionally, the circulation time was prolonged due to reduced macrophage phagocytosis [141]. 

Chowdhuri et al. synthesized a carboxymethyl chitosan-modified magnetic NMOF (IRMOF-3) composed of Zn^2+^ ions and 2-amino terephthalic acid with a target molecule, folic acid (FA), on its surface. The results displayed that the carboxymethyl chitosan increased the drug loading efficiency and improved the performance of the pH-responsive drug release. The anti-cancer drug Doxorubicin (DOX) was incorporated into the NMOF with a loading capacity of 1.63 g g^−1^. The release of the drug was investigated in two PBS media; one at a pH 7.4 and another containing an intercellular cancer cell environment at a pH 5.5, both at 37 °C. After 24 h about 26.72% of DOX was released at pH 7.4, whereas 55.1% of the drug was released at pH 5.5 [142]. Yang et al. modified Fe_3_O_4_ NPs with a layer of PVP and polyetherimide (PEI) to obtain Fe_3_O_4_@PVP-PEI nanospheres for the pH-responsive release of glycoproteins. Fe^3+^ ions and the organic ligand 1,4-phenylenebisboronic acid (PBA) were then added to produce the MOF nanocomposite Fe_3_O_4_@PVP-PEI@MOF-PBA with PBA also serving as a functional molecule in the MOF shell. This MOF-PBA shell displayed selective and pH-responsive capture (at pH 7) and release (pH 9) of glycoproteins [143]. 

## 5. BioMOFs for Drug Delivery Imaging Applications

Traditional therapeutics face challenges such as non-specific distribution, toxic side effects, poor pharmacokinetics and rapid clearance [144]. NP-based systems can overcome these challenges by providing a targeted delivery and high accumulation of the drug molecule. Doxil and Abraxane are commercialized nanodrugs that have opened doors to the development of other potential nanocarriers/drugs. The goal of creating a successful DDS is to achieve a high drug loading capacity with little to no side effects. 

**Figure 5:**
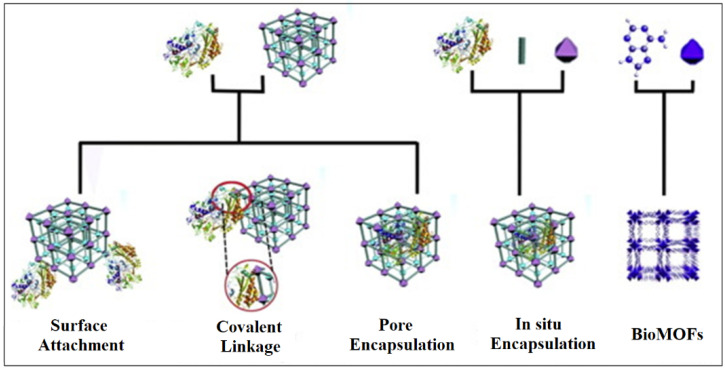
Various approaches of drug encapsulation/binding/within/onto MOF nanostructures. Reproduced with permission from Ref. [148]. Copyright © 2019, Elsevier.

A drug-MOF conjugate would combine the properties of both the biomolecule and MOF carrier and enhance the efficacy of the drug [145,146,147]. The MOF would create a stabilized microenvironment for the drug while improving its activity against harsh conditions, allowing for the separation and recovery of the drug upon internal/external stimuli. The formation of a drug-MOF conjugate could be synthesized using various methods (Figure 5). In general, biomolecules are incorporated through different methods [148]:

○ absorption into the pores of MOFs;
○ attachment on the external surface of MOF crystals;
○ in situ encapsulation into MOF crystals as ‘crystal defect’; and
○ directly used as ligands to synthesize MOFs.

In 2004 and 2005, Férey et al., developed mesoporous rigid chromium carboxylate MOFs (MIL-100 (Cr) and MIL-101 (Cr)) that were then loaded with the model drug, IBU [149,150]. The MOF structures possessed cage sizes around 25 to 34 Å and windows 5 to 16 Å. The Brunauer–Emmett–Teller (BET) surface areas (2100 to 4400 m^2^ g^−1^) allowed for a successful loading of IBU that was 4 times higher than silica materials and 9 times higher than zeolites [151,152]. Although mesoprous silica MCM-41 has a larger pore size and more impressive pore volume than MIL-101, the higher drug loading capacity in MIL-101 is evidence that the surface area plays an important role in drug encapsulation along with the drug/metal interaction [152]. The same group later encapsulated MIL-53 (Fe and Cr) with IBU and determined that the smaller the pore volume, the less drug loading occurred [153]. The release of IBU from MIL-100, MIL-101 and MIL-53 happened through diffusion and drug-matrix interaction. The MOFs released the drug cargo after 3, 6 or 21 days, respectively, when immersed in simulated body fluid (pH 7.4 @ 37 °C) [94]. 

Rojas et al. conducted an experimental and computational study on the physiochemical parameters that were driving the drug adsorption and desorption kinetics of aspirin and IBU using MIL-100(Fe), UiO-66(Zr) and MIL-127(Fe) [154]. Given that aspirin is hydrophilic and IBU is hydrophobic, the drug uptake was dependent on the cargo/matrix interaction and the accessibility of the drug in the framework. The release kinetics was dependent on: (i) the structure of the MOF, a slower release with a narrower pore or (ii) the hydrophobicity/hydrophilicity of the carrier (with UiO-66, aspirin displayed a faster release and slower release with IBU) [154].

**Figure 6:**
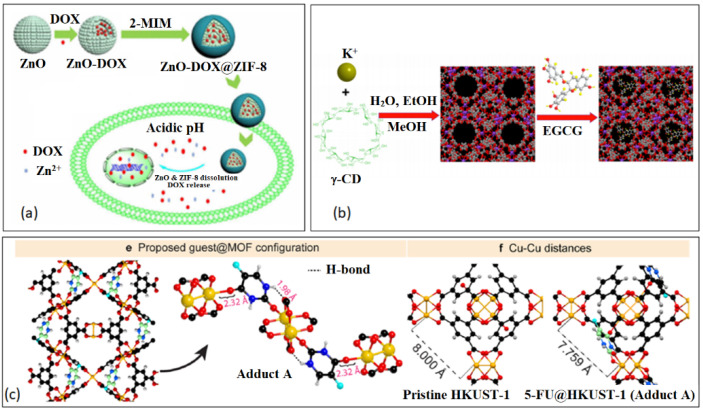
Examples of incorporation of drugs through of BioMOF nanostructures. (**a**) ZnO-DOX@ZIF-8. Reproduced with permission from Ref. [155]. Copyright © 2017, American Chemical Society. (**b**) Gallate-modified CD-MOF. Reproduced with permission from Ref. [158]. Copyright ©, 2019, Springer. (**c**) f-fluorouracil@HKUST-1. Reproduced with permission from Ref. [159]. Copyright © 2020, American Chemical Society.

In 2017, Zheng et al. developed a multifunctional hybrid nanosystem, ZnO-DOX@ZIF-8, consisting of mesoporous ZnO core and a microporous ZIF-8 shell. The drug, DOX, was loaded in the core and the shell was used to prevent a burst release of the drug at physiological conditions [155]. The ZnO-DOX@ZIF-8 core–shell NP displayed a 20% of loaded DOX release in buffer of pH 7.4 and over 80% in buffer of pH 5.5, making it a promising pH-responsive DDS, as shown in Figure 6a [155].

CD-MOF consists of six (α), seven (β) and eight (γ) glucopyranose units, a truncated cone with a hydrophobic inner pocket and a hydrophilic exterior. CD-MOF is a popular biomaterial because of its low cost, biodegradability, biocompatibility and very low toxicity. It has been utilized in various bio-applications, such as bio-imaging, bio-sensing, tissue engineering and drug delivery [156,157]. γ-CD-MOFs were later encapsulated with a model green tea, catechin, (-)-epigallocatechin gallate (EGCG), to protect the antioxidant from premature degradation [158]. CD-MOF-EGCG displayed a promising inhibitory effect on cancer cell growth of C6 cells and an enhanced antioxidant activity in basic solutions, as shown in Figure 6b [158]. 

More recently, researchers have combined porous MOFs with organic polymers to prevent a burst release of drug molecules [159]. Souza and colleagues synthesized a nanocomposite MOF, HKUST-1, embedded in a polymeric matrix (polyurethane) for the encapsulation and release of 5-fluorouracil (5-FU) (5-FU@HKUST-1/polyurethane). They used synchrotron microspectroscopy to track the release kinetics of 5-FU and discovered that HKUST-1 created hydrophilic channels within the hydrophobic polyurethane matrix to prevent a burst release effect. The MOFs role was to release the cancer agent while the polymer matrix protected the moisture sensitive MOF structure from water degradation, as shown in Figure 6c [159].

BioMOFs can also carry out multiple roles in therapeutic and diagnostic applications, such as drug delivery carriers and MRI contrast agents, simultaneously. MRI is a non-invasive imaging technique that provides 3D anatomical images based on the detection of nuclear spin reorientations. A contrasting agent, Gd, is usually given to patients intravenously to allow for faster proton alignment within the magnetic field, for a brighter image [160]. The Gd chelates a T_1_-weighted or positive signal enhancement and can help distinguish between diseases and non-diseased tissue [121]. MRI contrast agents exist as T_1_- (positive contrast), shortening the longitudinal relaxation time of water protons and T_2_- (negative contrast), which can reduce the transverse relaxation time of water protons [161]. BioMOFs can be utilized as a T_1_- or T_2_- contrast or combined for the use of drug delivery and as an MRI contrasting agent [161].

In 2006, NMOFs consisting of Gd^3+^ centers were synthesized using BDC and BTC linkers by reverse-phase microemulsions, which showed potential as contrasting agents for multimodal imaging [162]. The relaxation rates, R_1_ and R_2_, were enhanced because of the increased amount of Gd^3+^ centers present in the NMOFs [162].

MOF-based magnetic composites can also be used for targeted drug delivery as demonstrated by Ke et al. in 2011 [66]. The group synthesized the nanocomposites by encapsulating Fe_3_O_4_ nanorods in HKUST-1. The material displayed magnetic properties and high porosity, that was able to adsorb around 16 wt% of Nimesulide and release the drug for up to 11 days in physiological saline solution at 37 °C [66]. Later on, Pinna et al. developed a system for the magnetophoretic drug delivery of dopamine for Parkinson’s disease [163]. MIL-88A(Fe) crystals were grown around a polymer core containing superparamagnetic NPs with tunable sizes between 8 and 86 µm. The drug was stable and did not undergo oxidation within the carrier. The release of dopamine was assessed using spectrofluorimetry and showed a shorter burst effect (during the first 6 h in 1 mM PBS buffer at pH 7.4) and higher release efficiency when compared to silica-based carriers. Dopamine was directly taken up by PC12 cells, proving a targeted delivery effect [163]. A multifunctional Fe_3_O_4_@polyacrylic acid/Au nanoclusters/ZIF-8 NPs (Fe_3_O_4_@PAA/AuNCs/ZIF-8 NPs) was developed for the combination of tri-modal cancer imaging (magnetic resonance, computed X-ray tomography and fluorescence imaging) and drug delivery of DOX [164]. The goal was to develop a system that had the potential to be used as cancer treatment and diagnosis. The multifunctional NPs exhibited a DOX loading capacity of 1.55 mg per mg of NPs and a pH-responsive controlled drug release [164].

### 5.1. Stimuli-Responsive BioMOFs

**Figure 7:**
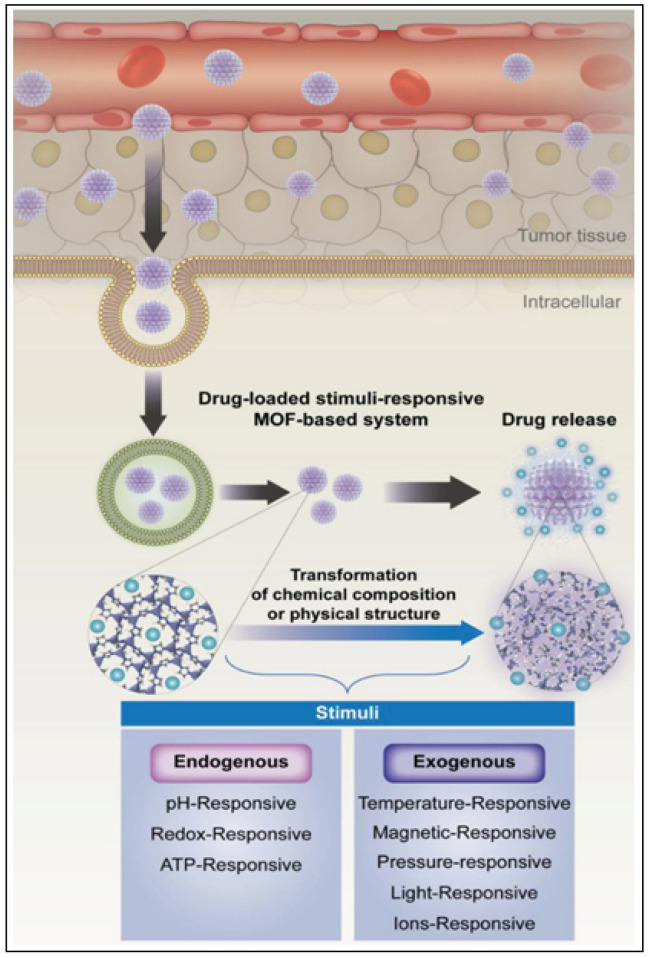
Various procedures of the development of stimuli-responsive BioMOF nanostructures, with examples of the types of stimuli. Reproduced with permission from Ref. [165]. Copyright © 2019, Wiley.

BioMOFs can be designed to respond to intrinsic triggers (pH, ATP, redox, etc.) and/or external triggers (temperature, ions, pressure, light) to offer an enhanced permeability and active targeting of the drug molecule, as illustrated in Figure 7 [165,166]. The DDS, once activated by these triggers, the drug molecule is released in a controlled manner, which is ideal for cancer treatment. 

#### 5.1.1. pH-Responsive BioMOFs

pH-responsive BioMOFs are of particular interest in cancer treatment as the coordination bonds are extremely sensitive to external pH changes [167,168,169,170]. BioMOFs can be designed to release cargo at tumor sites (pH ~ 6.5–6.9) for a targeted delivery and increased cellular uptake. ZIF-8, commonly used for the pH-responsive drug release, was encapsulated with DOX/Bovine serum albumin (BSA) NPs by Liang et al. [171]. The BioMOF carrier was designed for the protection of the drug as it is stable at pH7.4 and can decompose under acidic conditions and to also introduce positive charges on the outer surface for an increased cellular uptake [171,172,173,174]. The BSA/DOX@ZIF nanocomposite demonstrated a higher efficiency than the free drug and showed an improved biocompatibility when comparted to pure ZIF NPs [171].

The polyacrylic acid@ZIF-8 (PAA@ZIF-8) NPs are synthesized using a simple synthetic strategy for the ultrahigh DOX loading capacity of 1.9 g g^−1^ NPs [172]. This high drug loading capacity could be due to the electrostatic interaction between the positively charged drug molecule and the negatively charged –COOH groups located on the PAA@ZIF-8 structure. The coordination of Zn^2+^ and DOX also plays a role in the uptake of the drug molecule [172]. The DOX-loaded NPs were efficiently taken by MCF-7 cells and displayed a faster release of DOX in a mild acidic buffer solution (pH 5.5) when compared to a neutral PBS (pH 7.4). The nanocarriers showed low toxicity to normal healthy cells, making them a promising anti-cancer treatment and potential use in biological applications [172].

**Figure 8:**
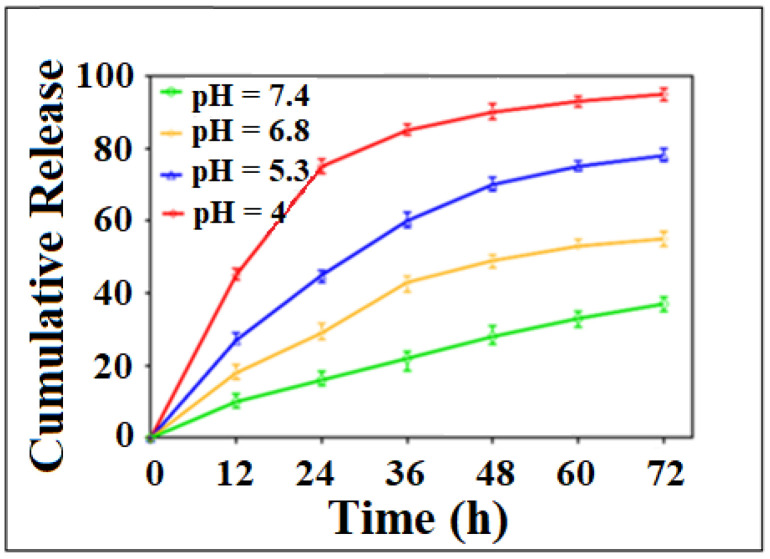
Cumulative release of 5-FU from 5-FU-loaded FOLA@NH_2_-Eu:TMU62. Reproduced with permission from Ref. [175]. Copyright © 2019, American Chemical Society.

Recently, Abazari et al. developed a luminescent FA amine-functionalized BioMOF (FOLA@NH_2_-Eu:TMU-62) for the delivery of the anti-cancer drug 5-FU [175]. Luminescent nanocarriers allow for the observation of structural specificities in tissues and cells and have been useful for drug delivery and bio-sensing [176,177]. They can be a promising tool for understanding biological processes, metabolism and pharmacokinetics and particularly useful for early stage cancer diagnosis and treatment [175,178]. The researchers in this study believed that the pH-responsive drug release of the carrier, along with the enhanced internalization of FA by the expression of the folate receptor expressing cells, would lead to a successful DDS for imaging and targeting. The pH-responsiveness of the system was studied using four difference pH values (pH 7.4, pH 6.8, pH 5.3 and pH 4). A miniscule amount of the drug was released at pH 7.4, however, about 55% of 5-FU was released at pH 6.8 and over 90% release with a buffer solution at pH 4.0 within 72 h (Figure 8). Decreasing the pH broke down the DDS and would most likely lead to a leakage of 5-FU from the carrier. Overall, the 5-FU-loaded FOLA@NH_2_-Eu:TMU62 carrier decreased tumor growth in MCF-7 cells and displayed a targeted delivery to cancer cells by means of the folate receptor and release of the drug in the cytoplasm [175].

#### 5.1.2. Ion-Responsive BioMOFs

A drug@MOF composite consists of strong electrostatic interactions between the ionic drug and ionic MOF structure [179,180]. This interaction allows for the release of the drug compound through diffusion, making it an ion-responsive mode of drug delivery. An et al. synthesized a porous anionic MOF, bio-MOF-1, using adenine as the building block for the storage and release of procainamide, a cationic antiarrythmic drug [181]. With a short half-life and a dosing of every 3–4 h, procainamide HCl, is an ideal drug for controlled release studies. After the drug was introduced in the MOF pores, through a cation exchange process over 15 days, the loading capacity was determined to reach up to 0.22 g g^−1^. The ionic interaction between the drug and the MOF triggered a release of the drug from the carrier when placed in PBS (pH 7.4). This was studied against a control (nanopure water) to prove that the drug released was mediated by the buffer cations [181]. 

**Figure 9:**
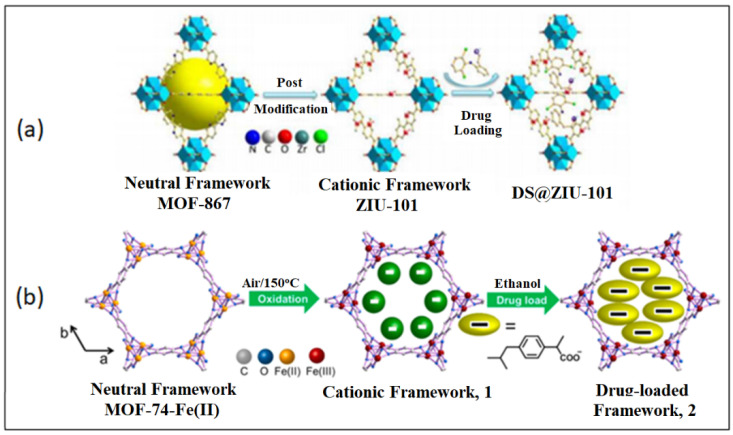
Examples of ion-responsive chemically modified BioMOF nanostructures. (**a**) Ionic-modified MOF-867. Reproduced with permission from Ref. [182]. Copyright © 2014, American Chemical Society. (**b**) Oxidative procedure of MOF-74-Fe(II). Reproduced with permission from Ref. [183]. Copyright © 2016, American Chemical Society.

Later on, the Hu group prepared a positively charged carrier, MOF-74-Fe (III) through the oxidation of the neutral MOF [182]. The cationic MOF was loaded with IBU anions and displayed a loading capacity of 0.19 g g^−1^. Two different release rates were observed due to the presence of coordinated or free IBU anions. The drug release occurred by diffusion and triggered by the anionic phosphate buffer solution. Therefore, the drug release can be controlled by regulating the carrier size and encapsulating them into other stimuli-responsive matrices (Figure 9a) [182].

In 2016, Yang and colleagues, constructed a cationic nanocarrier, ZJU-101, by post-modification of MOF-867. Methyl groups were added to the pyridyl groups of MOF-867 (zirconium with 2,2′-bipyridine-5,5′-dicarboxylate) and loaded with the anionic drug, diclofenac sodium [183]. Diclofenac sodium forms anions in solution, making it ideal for loading in cationic MOF pores. The loading of the drug was carried out in ethanol solution and determined to have a loading capacity of 0.546 g g^−1^. The drug demonstrated a more efficient release in PBS of pH 5.4 compared to PBS of pH 7.4. This displays a DDS that is pH-responsive and a drug release controlled by the anionic PBS and drug anions, as shown in Figure 9b [183].

#### 5.1.3. Magnetically Responsive BioMOFs

**Figure 10:**
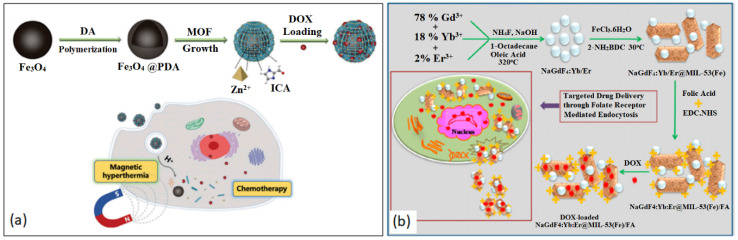
Examples of magnetically responsive chemically modified BioMOF nanostructures. (**a**) Fe_3_O_4_@PDA@ZIF-90 core–shell NPs. Reproduced with permission from Ref. [186]. Copyright © 2019, Taylor & Francis Online. (**b**) DOX-loaded NaGdF_4_:Yb/Er@MIL-53(Fe)/FA. Reproduced with permission from Ref. [187]. Copyright © 2020, American Chemical Society.

Magnetic-responsive DDSs work under the influence of a magnetic field and can be used for magnetic targeting, MRI, magnetic separation and magnetic hyperthermia [166,184,185]. In 2019, Chen et al. constructed a magnetic composite for the simultaneous treatment using magnetic hyperthermia and chemotherapy [186]. ZIF-90 was grown on polydopamine (PDA) coated Fe_3_O_4_ NPs to give Fe_3_O_4_@PDA@ZIF-90 core–shell particles with an average size of 200 nm. ZIF-90 was encapsulated with DOX with a loading efficiency of 80% (160 μg mg^−1^) due to the porosity and MOF/drug interaction. The Fe_3_O_4_ cores allowed for the localized temperature to reach hyperthermia conditions under an alternating magnetic field while eradicating tumor cells with an enhanced efficiency. This synergistic effect is a promising form of cancer treatment compared to magnetic hyperthermia alone, as shown in Figure 10a [186].

Recently, Mukerjee et al. designed a NP composite for theragnostic applications by doping NaGdF_4_ with Yb^3+^ and Er^3+^ NPs as imaging agents and MIL-53(Fe) as a drug carrier with FA conjugated on the surface for targeted drug delivery [187]. The nanocomposite was loaded with the anti-cancer drug, DOX, displaying a drug loading efficiency of 16% and drug encapsulation efficiency of 65%. Not only did the NaGdF_4_:Yb/Er@MIL-53(Fe)/FA system suppress tumor cell growth and enhanced cancer cellular uptake, it also showed colloidal stability and enhanced magnetic and fluorescence properties, making it an ideal candidate for both relaxation times, T_1_ and T_2_ MRI contrasting agents, as shown in Figure 10b [187]. 

#### 5.1.4. Temperature-Responsive BioMOFs

The ability of temperature-responsive MOFs to transform upon thermal stimuli while maintaining its crystalline structural integrity is of particular interest when it comes to designing drug delivery nanocarriers [180,188]. A temperature would induce a change in the thermoresponsive material allowing for the release of the drug cargo. Procainamide has recently been studied for the control release from UiO-66 in a temperature and pH sensitive environment [189]. The MOF was surrounded with *N*-isopropyl acrylamide (NIPAM) and acrylic acid (AA) by post-synthetic modification to give UiO-66-P(NIPAM-AA). PNIPAM is known for its thermoresponsive properties and its solubility in water at cloud point, making it useful in DDSs [190]. UiO-66-P(NIPAM-AA) experienced an on/off release when exposed to variations in pH and temperature. At pH 6.86 or low temperatures (less than 25 °C), the polymer composite turned into a coil conformation, allowing procainamide to be instantly released from the MOF pores. With a pH 4.01 or high temperatures (more than 40 °C), the polymer displayed a globular conformation and the release of the drug was suppressed. Therefore, drug release can be controlled by applying external stimuli (e.g., temperature or pH) even after the initial release of the drug from the MOF carrier [189]. 

Lin et al., synthesized Zn-GA loaded with the anti-cancer drug, methotrexate (MTX) by combining Zn(NO_3_)_2_·6H_2_O and L-glutamic acid using a one pot synthesis procedure [191]. The loading amount of MTX was estimated by proton nuclear magnetic resonance spectroscopy and was determined to have a drug loading capacity of 12.85%. The MTX@Zn-GA DDS exhibited much lower viability and increased cancer cell death with the introduction of the drug to PC12 cells. The DDS proved to have controlled release of the drug when triggered by pH or thermal stimuli without causing a burst release of the drug [191].

#### 5.1.5. Redox-Responsive BioMOFs

**Figure 11:**
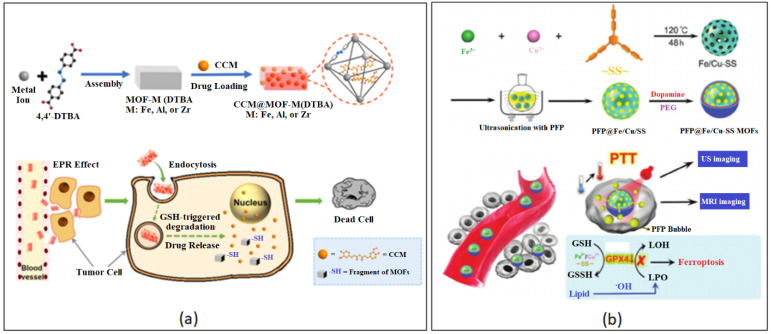
Examples of redox-responsive chemically modified BioMOF nanostructures. (**a**) CCM@MOF-M(DTBA). Reproduced with permission from Ref. [196]. Copyright © 2018, American Chemical Society. (**b**) PFP@Fe/Cu-SS MOFs. Reproduced with permission from Ref. [202]. Copyright © 2020, Wiley.

Redox concentrations vary between normal human tissue and cancerous tissue with tumors having a higher concentration due to the presence of reducing agents, such as glutathione (GSH) [192,193]. Redox-responsive BioMOFs [194,195] can be functionalized to target the receptor site that is responsible for the cleavage of the disulfide group in the presence of GSH. Lei and co-workers developed an intrinsic redox-responsive MOF carrier, MOF-M(DTBA) (M = Fe, Al or Zr) by using iron, aluminum or zirconium as metal nodes and 4,4′-dithiobisbenzoic acid (4,4′-DTBA) as the organic ligand [196]. DTBA is a GSH-sensitive organic ligand, which contains a disulfide bond, cleavable by GSH. In this study, the researchers loaded the drug carrier with the natural polyphenol anti-cancer drug, CCM producing, CCM@MOF-M(DTBA). The redox-responsiveness of the synthesized NP was exposed to various concentrations of DL-dithiothreitol (DTT). The results showed that with an increase in DTT in PBS, the release of CCM from the DDS was much faster (Figure 11a). It is clear that the cleavage of the disulfide bond led to a more efficient release of the drug molecule [196].

Ferroptosis is cell death dependent on iron and the accumulation of toxic lipid peroxides (LPOs), usually in tumor tissue. Once GSH is consumed by cells, glutathione peroxide 4 (GPX4) activity is inhibited and the level of lipid oxidation in cells increases accordingly, which leads to ferroptosis [197,198,199,200,201]. The ability to produce LPOs can lead researchers into developing effective targeted cancer treatment. Recently, a group synthesized a hybrid PFP@Fe/Cu-SS MOF by coordinating the disulfide-modified, phloroglucinol with Fe^3+^ and Cu^2+^ metals [202]. The porous MOF was loaded with perfluoropentane (PFP) and the nanocarrier was functionalized with PDA and PEG for improved stability and biocompatibility. The system proved to increase LPO concentration in tumor sites through redox reactions generating ·OH while inhibiting the activity of GPX4. This prevented the conversion of toxic LPO to nontoxic hydroxyl compounds in the presence of GSH (Figure 11b). The inclusion of the copper (II) metal also allows for the PFP@Fe/Cu-SS NP to be used as an MRI contrasting agent [202].

#### 5.1.6. ATP-Responsive BioMOFs

**Figure 12:**
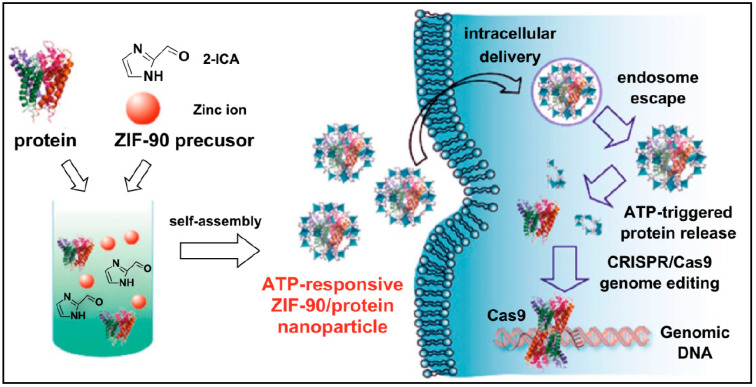
Examples of ATP-responsive chemically modified BioMOF nanostructures: ZIF-90/protein NPs. Reproduced with permission from Ref. [205]. Copyright © 2019, American Chemical Society.

Adenosine triphosphate (ATP) provides energy for all living cells by hydrolyzing phosphoanhydride bonds. Tumor cells contain a higher level of ATP compared to normal cells, allowing for the use of ATP-responsive DDSs. A number of BioMOFs have been used for the delivery of protein NPs that provide ATP response [203,204]. A ZIF-90/protein NP was synthesized using ZIF-90 as a platform for the cytosolic protein delivery and CRISPR/Cas9 genome editing. With ATP present as stimuli, the NPs were degraded to release the protein and this was due to the ATP and zinc metal bond of ZIF-90 [205]. The RNase A-NBC (RNase A modified with 4-nitrophenyl 4-(4,4,5,5-tetramethyl-1,3,2-dioxaborolan-2-yl) benzyl carbonate) protein, having cytotoxic effects against cancer cells, showed enhanced toxicity against HeLa cells when compared to the free protein (Figure 12). The cell viability was reduced to 15%, suggesting that ATP can promote protein release from ZIF-90/RNase A-NBC [205].

#### 5.1.7. Light-Responsive BioMOFs

**Figure 13:**
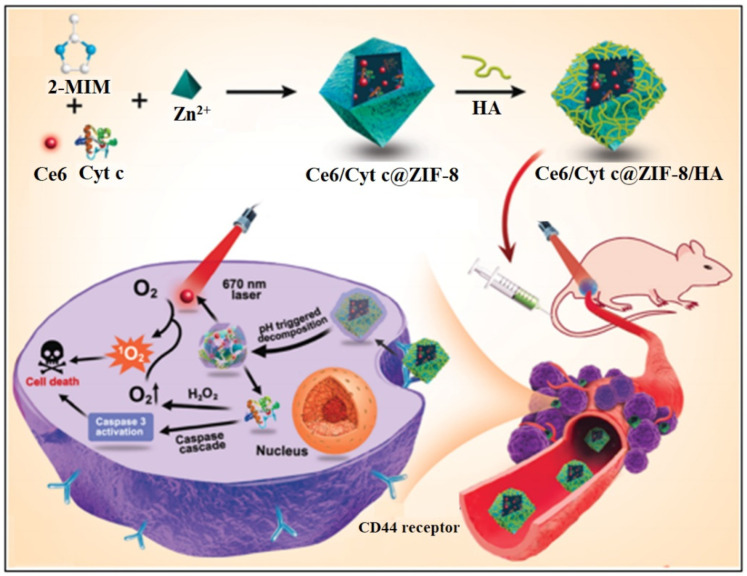
Examples of Light-responsive chemically modified BioMOF nanostructures: Ce6/Cyt *c*@ZIF-8/HA NPs. Reproduced with permission from Ref. [207]. Copyright © 2020, American Chemical Society.

Photodynamic therapy (PDT) is non-invasive and has been successfully used in treatment and diagnostics. Light-responsive MOFs can be designed to deliver biomolecules by irradiation of specific wavelengths of light where the photosensitizers (PSs) in target cells would absorb the light energy and produce reactive oxygen species (ROS) upon activation and killing the target cells [194,206]. Recently, ZIF-8 NPs were co-encapsulated with chlorin e6 (Ce6, a potent PS) and cytochrome *c* (Cyt *c*, a protein that induces apoptosis) by Ding et al. [207]. The NP was then functionalized with a hyaluronic acid (HA) shell to produce Ce6/Cyt *c*@ZIF-8/HA for targeted cancer cell activity. 1,3-Diphenylisobenzofuran (DPBF), an ROS probe, was used to determine the ROS generation of Ce6. Along with a pH-responsive release behavior of the nanocarrier, light irradiation caused Ce6 to produce ROS for a PDT effect. Furthermore, Cyt *c*, in the presence H_2_O_2_, will generate ROS while further inducing cell apoptosis (Figure 13). The co-encapsulation of therapeutic protein in the porous MOF structure allowed for a synergistic mode of cancer therapy that could lead to further developments in drug delivery design [207].

## 6. BioMOFs for Cancer Treatment 

MOFs have alleviated most of the limitations observed from traditional nanocarriers by providing an enhanced targeted delivery and higher accumulation of drug molecules [208,209]. Especially, MOFs can be tuned to respond to endogenous and external stimuli, which can be beneficial in cancer treatment and diagnosis. Their porosity and high surface area, allows for a more efficient loading of biomolecules while providing a low toxic carrier. Various cancer treatments and/or diagnosis using MOFs include radiotherapy, MRI imaging, carbon monoxide therapy, magnetic hyperthermia treatment and PDT depending on the targeted tumor cells. Table 1 outlines the applications of MOF-based nanomaterials for the treatment of different types of cancers. The following sections illustrate successful research trials in more details.

### 6.1. Breast Cancer

Breast cancer, according to recent statistics is the most prevalent cancer in women and the second most common overall, ranking the highest in Belgium, Luxembourg and the Netherlands [210]. Methods of treatment include radiotherapy, hormone therapy, chemotherapeutics and/or surgery, each having their disadvantages and toxic side effects. Breast cancer chemotherapeutics, such as Tamoxifen, can cause endometrial carcinoma and other unwanted side effects [211]. Using MOFs as anti-cancer drug delivery vehicles would reduce toxic side effects while increasing drug accumulation in breast cancer tissue compared to the free drug [48,212,213].

Zhang et al. engineered a triple-negative breast cancer (TNBC) targeted peptide (ZD2) using a single gold nanostar (AuNS) coated within MIL-101-NH_2_(Fe) producing a well-defined core–shell AuNS@MOF-ZD2 nanocomposite [32]. These nanocomposites were utilized for MRI and photothermal therapy (PTT) specifically towards TNBC. The AuNS@MOF-ZD2 nanoprobes targeted TNBC cells (MDA-MB-231) but not any other subtypes of breast cancer cells (MDA-MB-435, MDA-MB-468 and MCF-7), making them promising tools for theragnostic of breast cancers of a certain molecular classification [32]. Laha and co-workers also developed a system to target TNBC both in vitro and in vivo by encapsulating CCM in FA conjugated IRMOF-3 (IRMOF-3@CCM@FA) [33]. As previously mentioned in this review, folate receptors are overexpressed in tumor cells, allowing for the targeted delivery of drug molecules when FA is conjugated on the MOF surface. The IRMOF-3@CCM@FA system was successful in reducing tumor size in mice and induced apoptosis by upregulating the pro-apoptotic protein Bax and downregulating the anti-apoptotic Bcl-2 while upregulating JNK and p53 in human TNBC cells [33].

**Figure 14:**
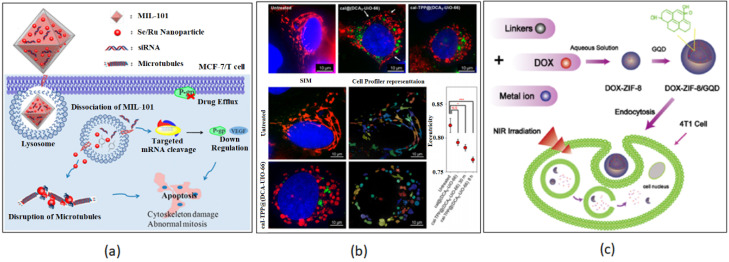
Applications of chemically modified BioMOF nanostructures for Breast Cancer treatment. (**a**) MIL-101. Reproduced with permission from Ref. [34]. Copyright © 2017, American Chemical Society. (**b**) UiO-66. Reproduced with permission from Ref. [35]. Copyright © 2020, American Chemical Society. (**c**) PDT/ZIF-8/Graphene Quantum Dots. Reproduced with permission from Ref. [40]. Copyright © 2017, American Chemical Society.

Microtubules (MT) play an important role in fundamental cellular activities, such as cell motility, cell division and intracellular trafficking. Evidence shows that a minor disruption in the dynamics of MTs can arrest cell cycle progression at mitosis and eventually lead to cell death. Current treatment includes Paclitaxel and Vinca alkaloids, which are designed to disrupt microtubule dynamics without changing MT mass for solid tumors and leukemias [34]. Although these drugs have been proven to be successful, tumor drug resistance can be caused by the overexpression of the multidrug resistance (MDR) protein, P-glycoprotein (Pgp, MRP4, ABCB1) and the class III β-tubulin (TUBB3) [214,215]. Breast cancer, in particular, has an overexpression of TUBB3, which can increase the dynamic instability of MTs, reducing the effect of taxane drugs [216,217]. The goal, to overcome this treatment obstacle, is to downregulate the expression of Pgp while disrupting the MT dynamics for the inhibition of drug-resistant tumor cells. Chen and co-workers developed a selenium/ruthenium NP-modified MIL-101(Fe) for the delivery of small interfering RNAs (siRNAs) to inhibit MDR genes while disrupting MT dynamics in MCF-7/T (taxol-resistance) cells. RNA interference can be useful for gene-targeted therapy because of its ability to suppress specific sequences in genes. Previous work has shown that the simultaneous delivery of nucleic acid drugs and chemotherapeutics reversed MDR in tumor tissue [218,219]. Selenium was chosen for its ability in reducing the incidence of cancers while having low toxicity [220,221,222,223]. In addition, ruthenium was added to the MOF NP for its anti-metastatic activity as an attempt for enhanced effect and efficiency [224,225]. MIL-101(Fe) was modified with cysteine owing to the strong linkage between Se/Ru and the MOF structure forming NPs (Se@MIL-101 and Ru@MIL-101). Small interfering RNA (siRNA)-loaded MOFs provided enhanced protection against MDR and nuclease degradation while increasing cellular uptake in MCF-7/T cells (Figure 14a). Furthermore, in vivo studies confirmed the chemotherapeutic efficiency of Se@MIL-101-(P+V)siRNA NPs by causing significant shrinkage of tumor size, nuclei fragmentation and chromosome condensation, and induced apoptosis [34].

More recently, researchers designed a mitochondria-targeted MOF that tested to increase the efficacy of the anti-cancer drug, dichloroacetate (DCA) when compared to the free drug [35]. Given that the mitochondria play an important role in oncogenesis, targeting it with a triphenylphosphonium (TPP) conjugated MOF would localize the DDS. Zirconium-based MOF, UiO-66, was conjugated with TPP and loaded with DCA, which inhibits pyruvate dehydrogenase kinase (PDK), shifting cancer cell metabolism from aerobic glycolysis to oxidative phosphorylation [226]. To confirm the targeted delivery to the site of action, Haddad et al. modified the particles with a fluorescent pyrene group, fTPP@(DCA5-UiO-66), for imaging and tracking (Figure 14b). It was reported that the required dose of the DCA5-TPP5-UiO-66 DDS was reduced to less than 1% when compared to the free drug (10%) [35]. 

Photodynamic therapy (PDT) involves three key components: (1) light (laser), (2) tissue oxygen and (3) the photosensitizer (PS) [227]. When the PS is illuminated using the appropriate wavelength, it is able to transfer the absorbed photon energy to oxygen molecules, generating reactive oxidative species (ROS) leading to cell death and tissue destruction [228,229]. Gold nanoclusters (AuNCs), as inorganic PSs, have been used in PDT, but due to their short circulation in the bloodstream their application is limited. To overcome this hurdle, Zhang et al. developed a stimuli-responsive ZIF-8 encapsulated with AuNCs and loaded with the anti-tumor drug, DOX to obtain AuNCs@MOF-DOX nanoprobes for breast cancer treatment. The structure of ZIF-8 degraded when exposed to a microenvironment with pH 5.5, releasing about 77.1% of DOX. The simultaneous treatment of DOX and PDT, displayed almost complete tumor inhibition and only partial inhibition when treated individually [39]. PDT using photo absorbers located in tumors can also be used to convert near-infrared (NIR) energy into heat, causing irreversible cellular damage leading to tumor eradication [230]. Tian et al. functionalized ZIF-8 with graphene quantum dots (GQDs) and encapsulated the carrier with the anti-cancer drug, DOX, using a one-pot synthesis method [40]. GQDs exhibit good NIR absorbance, high photothermal conversion efficiency, excellent thermal conductivity and low toxicity [231,232,233]. The ZIF-8/GQD multifunctional NPs were able to generate heat caused by NIR irradiation while also displaying a pH-responsive release of DOX under acidic conditions in breast cancer cells, 4T1 cells. The system exhibited a synergistic effect in cancer therapy and is a promising tool for future drug delivery design, as shown in Figure 14c [40].

### 6.2. Lung Cancer

According to the World Health Organization, lung cancer was the most common cause of cancer death worldwide in 2020 with around 1.8 million deaths [234]. Risk factors include environmental, lifestyle and occupational exposures with cigarette smoking being the lead cause of the deadly cancer [235]. About 16% of cases are detected before malignancy occurs with most detected during malignant stages [236]. Therefore, the development of a more complex nanocarrier with better diagnostic and therapeutic efficacy is needed [237,238]. Recently, Wang et al. modified a Fe-MOF system with a cationic polymer made from methyl viologen and polyallylamine hydrochloride (MV-PAH) and tested its effect on A549 lung cancer cells [53]. Methyl viologen, a bipyridyl herbicide, have genotoxic and cytotoxic effects due to its ability to generate ROS [53]. With the pH-responsiveness of the Fe-MOF system, the encapsulation of DOX and polyelectrolyte multilayer (PEM) coating, it was possible to successfully synthesize DOX@Fe-MOF@PEM NPs. The uptake of the NPs by A549 cells was successful and explained by the effective encapsulation of DOX due to the pH-sensitivity of the PEM coating. The amount of ROS generation in the cancer cells was 30-fold that of the control group and 8.29 fold that of the free drug alone. The DOX@Fe-MOF@PEM system induced higher apoptosis (62.9%) in A549 cells when compared with the Fe-MOF (3.34%), the free drug (22.39%) and DOX@Fe-MOF (39.79%) alone. These results prove the strong synergistic effect of the drug, Fe-MOF and PEM [53]. 

### 6.3. Liver Cancer

Hepatocellular carcinoma (HCC) and pancreatic ductal adenocarcinoma (PDAC) are the most deadly forms of cancer with the shortest life expectancy after diagnosis [239]. HCC is the most common primary cancer of the liver and the fastest rising cause of cancer related deaths in the US and second leading cause of cancer deaths in East Asia and Sub-Saharan Africa [240,241]. Both cancers are mostly inoperable and the primary treatment is chemotherapy or palliative procedures [239,242,243]. There have been limitations with targeted therapy and the only FDA approved drug, Sorafenib, is for the treatment of advanced HCC cases. Sorafenib, a protein kinase inhibitor, blocks vascular endothelial growth factor and platelet-derived growth factor receptors [244,245]. The epidermal growth factor (EGF) receptor kinase inhibitor, Erlotininib, is also used in conjunction with Gemcitabine to modestly improve the life expectancy in a sub-group of patients [246]. To overcome the hurdles associated with traditional chemotherapeutics, researchers are focused on developing combined cancer treatments to downregulate MDR, increase drug efficacy and reduce toxic effects. 

**Figure 15:**
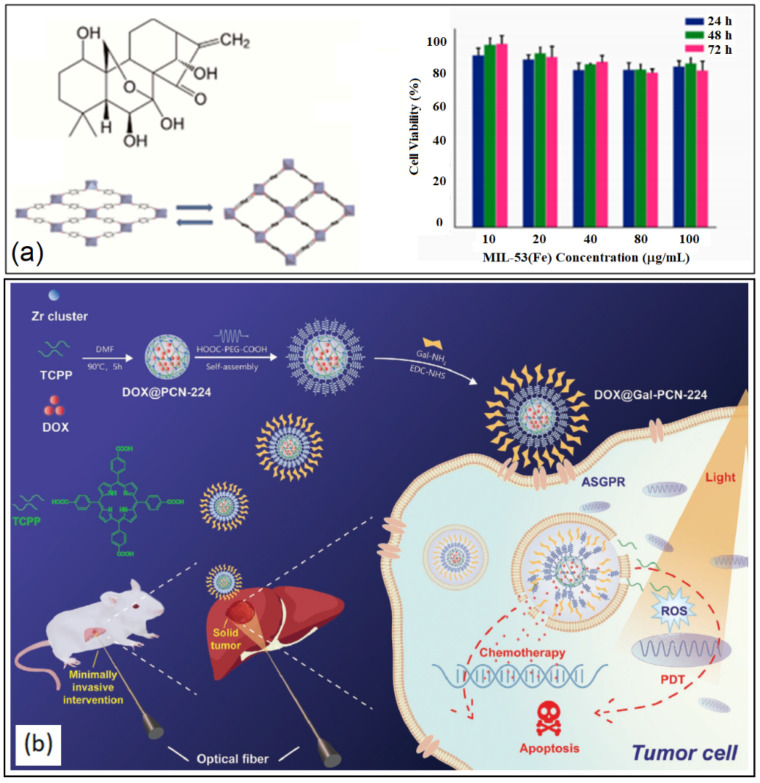
Applications of chemically modified BioMOF nanostructures for Hepatic Cancer Treatment. (**a**) MIL-53(Fe). Reproduced with permission from Ref. [59]. Copyright © 2018, MDPI. (**b**) DOX@Gal-PCN-224. Reproduced with permission from Ref. [65]. Copyright © 2020, Wiley.

Several BioMOFs have been explored as drug delivery vehicles for the treatment of HCC [247,248,249]. Leng et al. encapsulated the ent-kaurene diterpenoid compound, Oridonin (Ori), in MIL-53(Fe) for the delivery in human liver cancer cells, HepG2 [59]. Although possessing strong anti-cancer activity, Ori is moderately hydrophobic, chemically instable and has a short half-life [250,251]. Using the flexible, mesoporous and biocompatible MIL-53(Fe) would help alleviating the challenges of delivering the free drug on its own. The drug loading capacity was determined to be 56 wt% using a solvent diffusion method and left for 2 to 4 days at room temperature. The drug release was carried out in PBS pH 5.5 (91.75%) and pH 7.2 (82.23%) on the seventh day. Ori@MIL-53(Fe) showed inhibition of HepG2 cell proliferation at 28–57 µg/mL (equivalent to 15–30 µg/mL of free Ori), as shown in Figure 15a.

For an enhanced targeted delivery and improved pharmacokinetics, Chen and co-workers synthesized a Gd-porphyrin NMOF with the conjugation of FA to produce FA-NPMOF [62]. FA is used as a targeting ligand for specific drug delivery in tumor tissue while porphyrin MOFs work as PSs for their use in PDT. The addition of Gd to the nanostructure will provide the combination of imaging and therapy owing to Gd^3+^ ions having long electronic relaxation times. The study was conducted using HCC cells in *kras*^G12V^ zebrafish with DOX as the model drug for drug delivery. According to the MRI study, the T_1_-weighted signals were enhanced when FA-NPMOF dosage increased with no malformations observed. The tumor targeting effect of FA-NPMOF NPs on HCC-bearing *kras*^G12V^ zebrafish was determined by treating the cells with 200 µg/mL for 96 h and observing the fluorescence signal. A gradual increase in fluorescence occurred during the first 48 h suggesting that the NPs were specially delivered to HCC for that time period. There was also a noticeable shrinkage in tumor size with a tumor volume of around 23 mm^3^ in the FA-NPMOF/PDT group vs. 48 mm^3^ in the FANPMOF group, concluding the significance of using PDT in cancer treatments [62].

More recently, MIL-100(Fe) was synthesized by solvothermal method and tested on two types of hepatocytes: non-cancerous (HL-7702) and cancerous (HepG2) to determine the biocompatibility and safety of the MOF [63]. MTT assays on various concentration of MIL-100(Fe) on HL-7702 cells revealed a concentration less than 80 µg/mL was nontoxic with a cell viability greater than 85%. When the concentration was increased to 160 µg/mL, LDH was released indicating that the cell membrane was compromised and therefore, toxic to HL-7702 cells. With human liver cancer cells, Hep-G2, the cell viability was greater than 91% when treated with various concentrations of MIL-100(Fe), revealing a high tolerance up to 160 µg/mL. The study proved the use of MIL-100(Fe) as potential drug carriers in HCC treatment [63]. Sun and co-workers loaded a Gd(III) MOF carrier, [Gd(BCB)(DMF)](H_2_O)_2_, with an anti-cancer drug (5-FU) and evaluated its activity on both cell lines (HL-7702 and Hep-G2) [64]. The 5-FU-loaded MOF had a drug uptake of 36.4% and stimuli dependent release in an acidic cancer microenvironment. Furthermore, the drug-loaded carrier showed anti-cancer activity against HCC [63]. 

In 2020, Hu and co-workers synthesized a photosensitive porphyrinic galactose-modified MOF encapsulated with the anti-cancer drug, DOX (DOX@Gal-PCN-224) for the synergistic interventional PDT and chemotherapy using HCC cells and tumor tissue [65]. Galactose can target asialoglycoprotein receptor (ASGPR), which is expressed on the surface of liver cancer cells, enhancing the cellular uptake of the NP [252]. The Dox loading efficiency was determined to be around 14% while release studies revealed a 16% release of the drug in PBS (pH = 7.4) compared to an impressive 65% in a more acidic environment (pH = 5.6). Targeted cellular uptake was determined using confocal laser scanning microscopy and flow cytometry analysis. HepG2 and Huh7 cells exhibited significant fluorescence indicating active targeting using DOX@Gal-PCN-224 toward ASGPR+ cells. In vivo studies proved the DOX@Gal-PCN-224-RhB tumor targeting ability owing to the higher fluorescence intensity in tumor tissue compared to other organs. As for the combined chemotherapy and PDT effect on tumor growth inhibition, there was a noticeable increase (>40%) with the group treated using combination therapy, demonstrating the potential treatment for hepatocellular carcinoma, as shown in Figure 15b [65].

### 6.4. Colon Cancer

MOFs have also been used as cytosensors to detect colon (CT26) cancer cells [72,253,254,255]. Researchers created a nanohybrid nanoparticles by combining a Cr-based MOF (Cr-MOF) with cobalt phthalocyanine (CoPc). The idea of combining MOFs with metal nanoparticles can enhance the electrochemical features of the MOF and can be used in bio-sensing [256,257]. The early detection of colon cancer is paramount when treating patients as it is the third most prevalent cancer and has contributed to a high number of cancer-related deaths [258]. The Cr-MOF@CoPC cytosensor developed by Duan et al., demonstrated a higher sensing sensitivity towards CT26 cells when compared to the Cr-MOF and CoPc alone [72]. The low limit of detection in CT26 cells was 36 cells mL^−1^ and 8 cells mL^−1^ for electrochemical impedance spectroscopy (EIS) and differential pulse voltammetry, respectively. These values were compared with the detection limit in human normal L929 cells, which showed no significant EIS signals, proving the selectivity of the MOF towards CT26 cells.

A porous In(III)-based BioMOF, [In(Hpbic)(pbic)](DMF)_3_, was prepared using solvothermal synthesis with 2-(pyridin-4-yl)-1H-benzo[d]imidazole-5-carboxylic acid (H_2_pbic) as the organic linker for the treatment of SW60 colon cancer cells [69]. The nontoxic MOF was loaded with 36.2% of 5-FU and displayed a 73% cumulative release up to 192 h. Cell Counting Kit-8 assay revealed that the BioMOF system successfully reduced cell viability and proliferation in SW60 cells. Furthermore, the encapsulated MOF increased intracellular ROS levels with 65 and 96% of apoptosis at 1× and 3× IC_50_, respectively. Tumor volume was also inhibited in mice transplanted with SW60 colon cancer cells when treated with the 5-FU@[In(Hpbic)(pbic)](DMF)_3_ system [69]. More recently, Lv and coworkers synthesized an Er(III) MOF, [Er_3_(bpydb)_3_(HCOO)(OH)(H_2_O))]·6H_2_O_n_, using a rigid tripodal nitrogen-containing heterotopic ligand 4,4′-(4,4′-bipyridine-2,6-diyl) dibenzoic acid (bpydbH_2_) for the inhibition of Caco-2 colon cancer cells [70]. The Er(III) based MOF showed a significant decrease in cell viability, while the metal ion and ligand had no effect on the Caco-2 cancer cells. 

Endogenous H_2_S is found to be overexpressed in colon and ovarian cancers resulting from the catalysis of cysteine related enzymes [259,260,261,262,263]. When exposed to endogenous H_2_S, HKUST-1 NPs produce NIR-activatable copper sulfide for the synergistic PTT and CDT of colon cancer [71]. Researchers treated CT26 colon cancer cells with the Cu-based MOF as a stand-alone treatment. Not only can HKUST-1 be converted to photoactive copper sulfide for PTT, the MOF NP also exhibits a conversion of H_2_O_2_ in cancer cells into a more toxic **·**OH for CDT [71,264,265]. CT26 colon cancer cells exhibited a gradual reduction in cell viability with increasing concentrations of HKUST-1. 

### 6.5. Pancreatic Cancer

Pancreatic cancer is the seventh leading cause of cancer related deaths worldwide and the fourth in developed countries [266]. It remains one of the most lethal malignant neoplasms with over 400,000 new cases globally and a 5-year survival rate at only 9%. Several BioMOFs have been investigated as DDSs for the targeted delivery of drugs for the treatment of pancreatic cancer [267,268,269]. HKUST-1 was incorporated with Fe_3_O_4_ nanorods to produce magnetic MOF nanocomposites for the targeted drug delivery of Nimesulide in pancreatic cancer cells [66]. Nimesulide, a selective cyclooxygenase-2 (COX-2) inhibitor, exhibits chemopreventive activity by blocking COX-2, thereby decrease the concentration of prostaglandins inside tumor tissue. It has been shown to protect against *N*-nitrosobis(2-oxopropyl)amine-induced pancreatic tumors in hamsters and the post-initiation development of squamous cell carcinomas in 4-nitroquinoline-1-oxide-induced rat tongue carcinogenesis [270,271,272]. The Nimesulide carrier system demonstrated magnetic properties while showing a drug uptake of up to 0.2 g g^−1^. The system is a promising anti-cancer treatment as the drug displayed a sustained release for up to 11 days [66]. 

**Figure 16:**
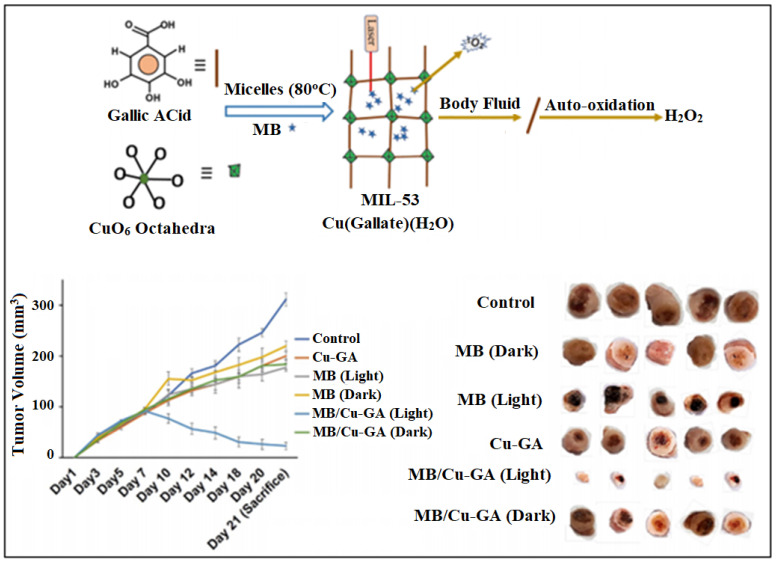
Cu-GA BioMOF Pancreatic Cancer Treatment. Reproduced with permission from. Ref. [67]. Copyright © 2019, American Chemical Society.

More recently, gallic acid (GA), an anti-oxidant and anti-cancer agent was used for the synthesis of a copper-gallic acid MOF (Cu-GA BioMOF) and post-synthetically loaded with the PS, methylene blue (MB), for PDT using Panc-1 cells [67]. The copper BioMOF framework was determined to have a BET specific surface area of 172 m^2^ g^−1^ and an average pore diameter of 2.2 nm. The loading efficiency of MB in the Cu-GA BioMOF reached 2 wt% owing to the hydrogen bonding between the nitrogen or sulfur groups on MB and the H^+^ of GA. There was a higher drug release of GA and MB (69% and 94%) when placed in PBS (pH 7, pH 4) making it ideal for the drug delivery in tumor tissue. The hydrophilic nature of GA will reduce its uptake into tumor tissue, whereas the Cu-GA BioMOF exhibits lipophilicity giving it the ability to interact with the cell membrane of tumor cells more readily than the free GA (Figure 16). According to the MTT assay using PANC-1 cells, Cu-GA BioMOF induced cytotoxicity (IC_50_ = 50 μg/mL) more efficiently than the free GA (IC_50_ = 25 μg/mL). The MB-loaded Cu-GA BioMOF induced a significant tumor growth inhibition in rats, proving its synergistic PDT and chemotherapeutic effects in pancreatic cancer cells [67]. 

### 6.6. Bladder Cancer

Bladder cancer occurs on the bladder mucosa when the cells’ DNA begins to mutate. The different types of bladder cancer include urothelial carcinoma, squamous cell carcinoma and adenocarcinoma. These types are differentiated by the type of cells that are affected. Risk factors include smoking, age, sex, chemical exposure, chronic bladder inflammation and family history [273]. CYLD (CYLD Lysine 63 Deubiquitinase) is a gene that plays a negative regulatory role in bladder cancer and the loss of CYLD expression can be observed in different types of human cancer [73,274]. MiR-181b (microRNA 181b), an RNA gene, has been shown to regulate the expression of CYLD, leading to the apoptosis of certain cancer cells [275]. 

Several types of BioMOFs have been tested as DDSs for the treatment of bladder cancer [276,277]. Wu et al. prepared a MOF based on Zn(II) via the rigid V-shaped ligand 2,6-di(2′,5′-dicarboxylphenyl)pyridine (H_4_L) with Zn(NO_3_)_2_·6H_2_O giving the complex, [Zn_3_(L)(OH)_2_(H_2_O)_4_](DMF)_5_ [73]. The BioMOF complex was observed for the detection of miR-130 and CYLD and their roles in the progression and downregulation of bladder cancer. The group exhibited a decrease in miR-130 and an increase in CYLD gene expression when treated with [Zn_3_(L)(OH)_2_(H_2_O)_4_](DMF)_5_. These results indicate that the MOF compound can induce programmed cell death by regulating the miR-130 and CYLD genes in bladder cancer.

### 6.7. Ovarian and Cervical Cancer

Ovarian cancer (OC) is the eighth most commonly occurring cancer in women and the deadliest among gynecological patients due to the asymptomatic nature of the disease [278,279]. Patient prognosis has not improved much compared to other cancers due to the resistance of epithelial ovarian cancer to platinum based chemotherapy [280]. Ovarian cancer patients are usually diagnosed with stage III and stage IV because of late detection and poor screening tests [281,282]. Current advanced ovarian cancer treatment involves a combination of surgical cytoreduction and chemotherapy [283]. The goal is to overcome drug resistance while using new imaging techniques and contrasting agents for early diagnosis and targeted delivery towards ovarian cancer cells [284]. Silencing genes via siRNA, has been used in combating resistant cancers and can reverse cisplatin (Cis) resistance in ovarian cancer [285,286,287,288]. 

**Figure 17:**
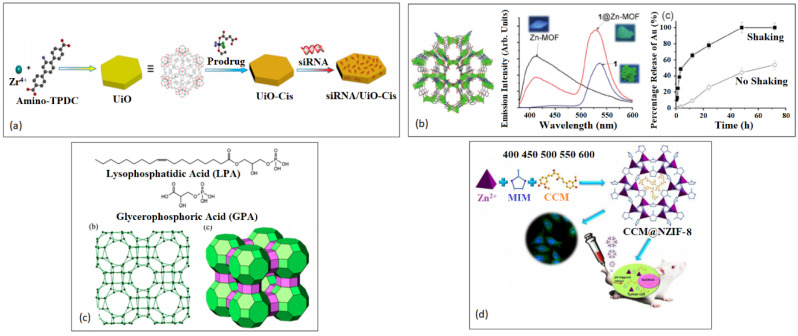
Applications of chemically modified BioMOF nanostructures for Ovarian (**a**,**b**) and Cervical (**c**,**d**) Cancer treatment. (**a**) siRNA/Cis/UiO. Reproduced with permission from Ref. [74]. Copyright © 2014, American Chemical Society. (**b**) Zn-MOF. Reproduced with permission from Ref. [76]. Copyright © 2018, Wiley. (**c**) Eu Tb-ZMOF. Reproduced with permission from Ref. [75]. Copyright © 2015, American Chemical Society. (**d**) CCM@NZIF-8. Reproduced with permission from Ref. [83]. Copyright © 2015, American Chemical Society.

Researchers have recently considered several BioMOFs for the encapsulation of drugs aimed at the treatment of OC [289,290]. He et al. encapsulated a UiO NMOF with siRNA and the anti-cancer drug, Cis, for the co-delivery in human ovarian cancer cells, SK-OV-3 [74]. The nanocarrier protects the siRNA from nuclease degradation allowing for an increased cellular uptake while promoting release from endosomes for the silencing of MDR genes in OC cells. UiO-Cis exhibited a 12.3 wt% drug loading capacity determined by inductively coupled plasma mass spectrometry. Dynamic light scattering measurements increased after the loading of siRNA confirming its presence in the DDS. There was a much higher siRNA/UiO-Cis cellular uptake compared to the free siRNA solution confirmed by confocal laser scanning microscopy with red fluorescence in the cytoplasm of SK-OV-3 cells. The nanosystem was successful in the knockdown and reversal of three MDR- relevant genes (survivin, Bcl-2 and P-gp) with IC_50_ decreasing by more than 11-fold by co-delivering pooled siRNAs and cisplatin in a NMOF carrier, as shown in Figure 17a [74].

Sun et al. reported a dinuclear gold(I) pyrrolidinedithiocarbamato (PDTC) complex with a bidentate carbene ligand for the cytotoxic activity towards Cis-resistant ovarian cancer cells, A2780cis [76]. PDTC, a dithiocarbamate, has been proven to exhibit cytotoxic and antiangiogenic activities [291]. Furthermore, metal-based dithiocarbamato complexes have been proven to have anti-cancer potencies comparable to Cis [292]. Zn-based BioMOF (zinc(II), adenine and a BTC linker was used as a carrier for the uptake and release of dinuclear gold(I) pyrrolidinedithiocarbamato complex. The Zn-MOF complex was successful in killing A2780cis cancer cells with a decreased cell survival by 50% when the co-incubation period was greater than 24 h. The antimigratory activity of the Zn-BioMOF complex was exhibited using a transwell antimigratory assay where the Zn-MOF complex effectively inhibited A2870cis OC cells, as shown in Figure 17b [76].

Lysophosphatidic acid (LPA), a bioactive phospholipid, causes the proliferation of cancer cells with elevated levels in plasma, suggesting that it plays an important role in the pathophysiology of cancer cells [293,294]. LPA has also been shown to alter receptor expression in ovarian carcinogenesis and metastasis when compared to other epithelial tumors; therefore, the early detection of LPA levels in plasma could aid in early diagnosis and treatment of the disease [295,296,297]. Zhang et al. constructed a three mixed-crystal isostructural MZMOFs with variable Eu:Tb stoichiometry for the detection of LPA, the biomarker for OC [75]. Lanthanide-MOFs exhibit luminescent properties associated with those of lanthanide cations, which can be tuned by host-guest chemistry for the chemical sensing of LPA [298]. MZMOF-3 (Eu_0.6059_Tb_0.3941_-ZMOF) was successful in the detection of LPA in the presence of other major compounds in the blood plasma making it a promising biochemical sensing tool, as illustrated in Figure 17c [75].

Recently, Chen et al. loaded a nucleic acid functionalized UiO-68 with DOX for the unlocking and release of the anti-cancer drug towards OVCAR-3 ovarian cancer cells [80]. The nucleic acid includes a base sequence that is complementary to the miRNA-221, a specific biomarker for ovarian cancer cells, inducing the ‘un-locking’ of the BioMOF carrier for targeted delivery of DOX [299]. The research revealed the enhanced release of DOX from the carrier with increased concentrations of miRNA-221, proving its unlocking capabilities in target tissue when exposed to exonuclease III. Upon treatment with the DOX-loaded miRNA-221-responsive NMOFs, OVCAR-3 cells exhibited a 50% decrease in cell viability, displaying cytotoxicity towards OCCAR-3 ovarian cancer cells [80].

More recently, a Cu(II)-based BioMOF, [(Cu(L)_2_(H_2_O)_2_](DMF)_4_)_n_ (L = 3-(1*H*-pyrazol-4-yl)pyridine) was studied for its inhibitory effect on Hey ovarian cancer cells [81]. MTT assay, CCK-8 proved the anti-cancer activity of the MOF system with an IC_50_ value 2.81 ± 0.17 μg/mL. The system induced cell apoptosis in Hey cells by increasing ROS accumulation. The treatment was dose-dependent, meaning that the level of ROS accumulation increased significantly with increased concentrations of the Cu-BioMOF. 

Cervical cancer is the fourth most common gynecologic cause of cancer with about 99% of cases linked with high-risk human papillomaviruses (HPV). Early diagnosis and treatment can lead to very successful eradication of the cancer [300,301]. BioMOFs have been also investigated as DDSs in this regard [302,303]. Zheng and co-workers synthesized a CCM-loaded nanoscale ZIF-8 (CCM@NZIF-8) NP to evaluate the antitumor effect on xenograft tumors of U14 cervical cancer [83]. The CCM@NZIF-8 NPs exhibited a drug encapsulation efficiency of 88.2% and a tumor inhibitory rate of 85% making it an ideal, biocompatible drug delivery carrier. The NPs were also proven to be highly stable when placed in methanol solution, PBS and fetal calf serum solutions as the hydrodynamic parameters did not change significantly. TGA was also used to measure the stability of the NP, resulting in the structure breaking down at 547 °C. Furthermore, CCM@NZIF-8 had a higher inhibition rate and enhanced cytotoxicity in HeLa cells when compared to the free CCM due to the effective endocytosis by the cells, as shown in Figure 17d [83]. 

### 6.8. Oral Cancer

Traditional single cancer therapy has limitations and harmful side effects, owing to the need of multimodal systems for a more effective therapy [304]. BioMOFs and their nanocomposites have been investigated in this regard [305,306]. Xiang et al. synthesized magnetic MOF NPs with porous carbon (Fe_3_O_4_@C) for the combined cancer therapy and magnetic-triggered hyperthermia in human oral squamous cell carcinoma cell line, CAL27 and CAL27 tumor-bearing mice [58]. The NPs were further coated with PVP and encapsulated with DOX to give Fe_3_O_4_@C-PVP@DOX nanocomposites. About 70% of DOX was adsorbed and loaded in the porous MOF NPs with only 4% being released after 6 h at pH 7.4, indicating the effectiveness of the drug loading. The DOX release increased when an alternating magnetic field (AMF) was added, proving that the NPs are magnetically triggered. Furthermore, the NPs were incubated with CAL27 cells followed by magnetic hyperthermia (MHT) at 43 °C where more DOX was released, indicating the AMF-triggered heat leads to the accumulation of the drug towards cancer cells [58]. 

Tan and researchers developed a hybrid nanocomposite (DOX/Cel/MOFs@Gel) by integrating IRMOF-3 with a thermosensitive hydrogel, poly(D,L-lactide-coglycolide)-poly(ethylene glycol)-poly(D,L-lactide-coglycolide) triblock copolymers (PLGA-PEG-PLGA) where DOX and celecoxib (Cel) were coloaded for a localized treatment in KB and SCC-9 oral cancer cells [57]. The group compared the nanocomposite along with the free drug, DOX-BioMOFs and DOX/Cel-BioMOFs. DOX exhibited more than 80% release in an acidic environment (pH ~ 6.5) with a sustained release due to the protective layer from the IRMOF-3. The introduction of the thermosensitive hydrogel decreased the burst release of Cel from the nanocomposite and about 66% of the drug experienced a cumulative release after 11 days in the acidic medium, which could be due to the hydrophobic nature of the gel [57]. The cytotoxicity studies on KB and SCC-9 oral cancer cells revealed that DOX/Cel-BioMOFs had the highest amount of cell death. The thermosensitive gel added an extra layer for the drug to be able to break through, leading to a weakened cell killing effect. However, the DOX/Cel-BioMOFs@Gel nanocomposite exhibited the most tumor ablation in nude mice bearing SCC-9 xenografts. This could be due to the steady drug release and the combined effect of the DOX and Cel, indicating a localized treatment for oral cancer patients. 

### 6.9. Brain Cancer

Brain cancer is one of the most aggressive cancers due to late diagnosis and the inability of DDSs being able to pass through the blood–brain barrier (BBB). NPs have tremendously improved early and accurate diagnosis while providing enhanced sensitivity and targeted drug delivery [307,308,309,310,311]. Researchers studied the effect of a planar MOF-based composite on U87MG brain cancer cells and U87MG tumor-bearing nude mice [85]. They seeded Au NPs on Zr-based porphyrinic BioMOF nanosheets and loaded them with _L_-Arg for PDT and gas therapy [85]. The Au NPs were added for their ability to catalyze glucose into H_2_O_2_ and gluconic acid in the presence of O_2_ [312,313]. This generated H_2_O_2_ can metabolize L-Arg to L-citrulline, leading to NO generation [314,315,316]. NO could inhibit cancer growth by causing DNA damage, the nitrosylation of certain enzymes or mitochondrial ablation [317,318,319]. A hydrogen peroxide assay kit was used to measure H_2_O_2_ generation and the results indicated that the levels of H_2_O_2_ decreased due to the consumption by L-Arg. Griess assay revealed that NO generation was proportional to L-Arg loading and that the designed MOF composite (GMOF-LA) could produce NO in the presence of converted H_2_O_2_, leading to an enhanced tumor suppression by means of a biocatalytic cascade [85]. The cellular uptake of the MOF composite in U87MG human glioblastoma cells increased gradually with a maximum internalization reaching 12 h. Furthermore, the GMOF nanosheets showed little to no toxicity against U87MG cells when compared to the combination treatment system (GMOF-LA + laser). The cell viability decreased to 18.6% when the MOF composite was used in conjunction with PDT and NO-mediated gas therapy (GMOF-LA). Finally, in vivo studies revealed the accumulation of GMOF-LA nanosheets in U87MG tumor-bearing mice reaching a maximum value of 4.45 ± 0.70%ID g^−1^, owing to the enhanced permeability and retention effect [85]. This multifunctional MOF composite can pave the way for future developments using nanoreactor-mediated therapy. 

### 6.10. Blood Cancer

Leukemia, a cancer of blood tissue, occurs when bone marrow overproduces white blood cells, causing an abnormal amount white blood cells leading to overall malfunction. Risk factors include genetics, smoking, history of cancer treatment, chemical exposure and family history [320]. Traditional DDSs used for treating leukemia revealed some challenges such as stability, drug leakage and toxicity [321,322,323,324].

A porous MOF was synthesized by reacting 3-phenylpyridine polycarboxylic (H_3_L) ligands with Ni(NO_3_)_2_·6H_2_O giving, (Me_2_NH_2_)[Ni_3_(L)_2_(μ_3_-OH)(H_2_O)]·2DMF [86]. DCFH-DA detection kit assay was used to determine ROS production of the MOF compound in HL-60 promyelocytic leukemia cells. The results revealed ROS accumulation in a dose-dependent manner, with 58.70% and 90.02% at 1× IC_50_ and 3× IC_50_, respectively. The compound was further tested on HL-60 cells using the MTT assay. The results showed significant reduction in cell colonies and cell viability with IC_50_ of 2.13 ± 0.07 µg/mL, suggesting the BioMOFs anti-cancer effect without the addition or encapsulation of other drug compounds. 

## 7. Conclusions and Outlooks

MOF materials offer great potential in drug delivery and theragnostic applications due to their high drug loading capacities, ease of functionalization, biocompatibility and high flexibility. Significant progress has been made using these hybrid systems, not only as drug carriers but also for magnetophoretic therapy and as diagnostic tools. BioMOFs synthesized from non-toxic metals and endogenous linkers would further reduce unwanted side effects while improving efficacy of therapeutic agents. Additionally, using metals, such as copper and zinc, can give the DDS the added benefit of having antimicrobial properties. The functionalization of the MOF particles with polymer and lipid bilayer coatings have improved pharmacokinetics tremendously while the incorporation of siRNA in the carrier can overcome MDR in diseases that are drug resistant. The various methods of synthesizing BioMOF composites (solvothermal, sonochemical, reverse-phase microemulsion, etc.) allows for numerous administration routes and formulations [94]. Despite recent advances in BioMOF systems and their use as drug delivery carriers, further in vivo studies need to be conducted to understand the metabolic mechanisms and the pharmacokinetics of BioMOFs in various organs and tissues and to dissect the degradation pathway of the MOF structure and the kinetics of drug delivery. Our group has recently observed that the stability of some BioMOFs in PBS is poor when compared to RPMI culture media. It is critical to dive deeper in studying the decomposition and stability of MOF materials as this will give us more insight on the accumulation of the nanocarrier systemically. More recently, BioMOFs have been used as stand-alone treatment against certain cancers. A treatment plan using a biocompatible and biodegradable MOF without the need of chemo drugs will provide a much safer option for cancer patients. 

## Figures and Tables

**Table 1:** Applications of MOF-based nanomaterials for the treatment of different types of cancers.

MOF	Cancer	Targeting Material/Drug	Cell Lines	Activity	Ref
MIL-101-NH_2_(Fe)	Breast	single gold nanostar (AuNS), targeted peptide (ZD2)	MDA-MB-231	MRI, photothermal therapy	[111] (Zhang)
IRMOF-3	Breast	curcumin (CCM), folic acid (FA)	MDA-MB-468, 4T1	ROS-mediated DNA, mitochondrial DNA damage	[112] (Laha)
MIL-101(Fe)	Breast	selenium/ruthenium nanoparticles, siRNAs	MCF-7/T	instability of MTs and disruption of mitotic spindle formation	[113] (Chen)
UiO-66	Breast	triphenylphosphonium (TPP), dichloroacetate (DCA)	MCF-7	mitochondria-targeted	[126] (Haddad)
Fe_3_O_4_@UiO-66-NH_2_	Breast	Fe_3_O_4_, DOX, highly fluorescent carbon dots (CDs), nucleolin-binding aptamer, AS1411	MDA-MB-231	cellular bioimaging and chemotherapy	[200] (Alijani)
ZIF-8	Breast	AuNCs, DOX	-	photothermal therapy, chemotherapy	[131] (Zhang)
ZIF-8	Breast	graphene quantum dots (GQDs)	4T1	Photothermal therapy, chemotherapy	[133] (Tian)
CS/Zn-MOF@GO	Breast	chitosan (CS), graphene oxide (GO), 5-Fu	MDA-MB 231	chemotherapy, pH sensitive	[193] (pooresmail)
Zr-Fc MOF Nanosheet	Breast	ferrocene-based MOF (Zr-Fc MOF) nanosheet	4T1	Photothermal therapy and chemodynamic therapy	[210] (Deng)
MD@Lip	Breast	dichloroacetic acid, (DCA), Fe(II) MOF	MDA-MB-231	ROS chemotherapy	
Zr-based porphyrinic MOF	Breast, Lung	porphyrin, DOX, indocyanine green (ICG)	4T1, A549, U87MG	photothermal therapy, chemotherapy	[191] (Sun)
ZIF-67	Breast	phosphorus nanosheets (BPNSs), ferrocene (Fc), indium tin oxide (ITO) slice, methylene blue (MB)-labeled single- strand DNA aptamer	MCF-7	aptasensor for detecting specific cancer cell-derived exosomes	[192] (Sun)
porphyrin Pd-MOF	Breast	Pd, porphyrin	4T1, HeLa, 4T1 tumor-bearing mice	hydrogenothermal chemotherapy, photoacoustic imaging	[190] (Zhou)
porphyrin MOF	Breast	porphyrin-like single atom Fe(III) centers, DOX	MCF-7	photodynamic therapy, photothermal therapy, photoacoustic imaging	[188] (Wang)
HKUST-1	Breast	Cu^2+^, Vk3	4T1	chemodynamic therapy	[189] (Tian)
Cu-MOF/Ce6	Breast	Cu^2+^, chlorin e6 (Ce6)	MCF-7	chemodynamic therapy, sonodynamic therapy	[207] (Zhang)
N_3_-bio-MOF-100	Breast	CCM, FA	4T1	chemotherapy, pH sensitive	[202] (Alves)
Tb-MOF-on-Fe-MOF	Breast	Bimetallic FeTb-MOFs	MCF-7	aptasensor for detecting CA125	[184] (Wang)
TA-Fe/ART@ZIF-8	Breast	artemisinin (ART), tannic acid (TA), Fe(II)	MDA-MB-231, MDA-MB-231 xenograft tumors	chemotherapy	[206] (Li)
HA-PCN	Breast	PCN-224, HA, DOX	MCF-7/MDR	photodynamic therapy, chemotherapy	[208]
La(III)-MOF	Breast, Lung	La(III)-MOF (fluorophore) Ag NPs (quencher), 5′-amino-labeled ssDNA strands (aptamers)	miRNA-155 (biomarker)	photoluminescence Quenching-Based Detection of miRNA-155	[195] (Afzalinia)
Fe-MOF@PEM	Breast, Lung	polyelectrolyte multilayer (PEM), DOX	MCF-7, A549	ROS, chemotherapy	[197] (Wang)
Gd-MOF	Lung	5-Fu	A549	chemotherapy	[198] (Wei)
IRMOF-3	Oral	thermosensitive hydrogel, DOX, celecoxib (Cel)	KB, SCC-9	Chemotherapy (pH responsive	[185] (Tan)
Fe_3_O_4_@C nanocomposite	Oral	Fe_3_O_4_, DOX	CAL27	MRI-guided magnetic-triggered hyperthermia and chemotherapy	[196] (Xiang)
MIL-53(Fe)	Hepatic	oridonin (Ori)	HepG2	chemotherapy	[144] (Leng)
DHA@ZIF-8	Hepatic	Dihydroartemisinin (DHA)	HepG2, SMMC-7721, BEL-7404	chemotherapy	[194] (Li)
Fe^2+^ doped ZIF-8	Hepatic	Ferrous ion, DHA	HepG2	chemotherapy	[203] (Xiao)
porphyrin MOFs	Hepatic	FA, gadolinium (Gd)	HepG2, L02	photodynamic therapy, MRI	[147] (chen)
MIL-100(Fe)	Hepatic	-	HepG2	chemotherapy	[148] (chen)
Gd(BCB)(DMF)](H_2_O)_2_	Hepatic	5-Fu	Hep-G2	chemotherapy	[149] (Sun)
PCN-224	Hepatic	Galactose, DOX	HepG2, Huh7	Interventional photodynamic therapy, chemotherapy	[150] (Hu)
HKUST-1	Pancreatic	Fe_3_O_4_ nanorods, Nimesulide	-	chemotherapy	[81] (ke)
Cu-GA NMOF	Pancreatic	methylene blue	Panc-1 cells	chemotherapy, photodynamic therapy	[156] (Sharma)
UiO-Cis	Ovarian	siRNA, cisplatin	SKOV3 cells	chemotherapy	[168] (He)
Ln-ZMOFs	Ovarian	Terbium (Tb), Europium (Eu)	LPA (biomarker)	Biochemical sensing	[174] (Zhang)
Zn-MOF	Ovarian	dinuclear gold(I) pyrrolidinedithiocarbamato	A2780, A2870cis, HepG2, U-87 MG, MDCK	chemotherapy	[176] (Sun)
HKUST-1	Ovarian	Cu-MOF	SKOV3	chemotherapy	[199] (Chen)
MIL-88A	Ovarian	minicircle DNA (MC), MC encoding anti-CD3/anti-EpCAM bispecific T cell engager (MC.BiTE)	SKOV3	Bispecific T-cell engager (BiTE) immunotherapy	[201] (Zhao)
FeN200@GOx@M	Ovarian	FeN, glucose oxidase (GOx)	A2780	chemotherapy	[204]
UiO-68	Ovarian, Breast	complementary sequence to (miRNA)-21 or miRNA-221, DOX	OVCAR-3, MCF-7	miRNA responsive, chemotherapy	[179] (Chen)
Cu-MOF	Ovarian	-	Hey ovarian cancer cells	chemotherapy by ROS accumulation	[181] (Li)
NZIF-8	Cervical	CCM	HeLa, xenograft tumors of U14	chemotherapy	[183] (Zheng)
NH_2_-MIL-88B (Fe)	Cervical	Chloroquine (CQ)	cervical carcinoma cell line HeLa, A375	nanocatalytic therapy, ROS-induced oxidative damage	[186] (Yang)
HKUST-1	Colon, Cervical	Cu^2+^	CT26, CT26 tumor-bearing mice, human cervix cancer cells HeLa	H_2_S-activated photothermal therapy, chemodynamic therapy	[187] (Li)
[Zn_2_(L)(H_2_O)_2_](DMA)_2_	Colon		SW60	chemotherapy	
In^III^-MOF	Colon	5-Fu	SW60	ROS, chemotherapy	[205] (Li)
Cr-MOF@CoPc	Colorectal	cobalt phthalocyanine (CoPc) NPs	CT26	biosensing	[209] (Duan)
Ni(II) MOF	Leukemia		HL-60	ROS, chemotherapy	[207] (Xi)

## Data Availability

Data presented in this review are based on a screening of the literature. All data are related to their original manuscripts.
